# Biohybrid Micro/Nanorobots: Pioneering the Next Generation of Medical Technology

**DOI:** 10.1002/adhm.202402102

**Published:** 2024-10-07

**Authors:** Atefeh Zarepour, Arezoo Khosravi, Siavash Iravani, Ali Zarrabi

**Affiliations:** ^1^ Department of Research Analytics Saveetha Dental College and Hospitals, Saveetha Institute of Medical and Technical Sciences, Saveetha University Chennai 600 077 India; ^2^ Department of Genetics and Bioengineering, Faculty of Engineering and Natural Sciences Istanbul Okan University Istanbul Turkiye 34959; ^3^ Independent Researcher W Nazar ST, Boostan Ave Isfahan Iran; ^4^ Department of Biomedical Engineering, Faculty of Engineering and Natural Sciences Istinye University Istanbul Turkiye 34396; ^5^ Graduate School of Biotechnology and Bioengineering Yuan Ze University Taoyuan 320315 Taiwan

**Keywords:** biohybrid micro/nanorobots, biomedical applications, diagnostics, targeted drug delivery, tissue engineering

## Abstract

Biohybrid micro/nanorobots hold a great potential for advancing biomedical research. These tiny structures, designed to mimic biological organisms, offer a promising method for targeted drug delivery, tissue engineering, biosensing/imaging, and cancer therapy, among other applications. The integration of biology and robotics opens new possibilities for minimally invasive surgeries and personalized healthcare solutions. The key challenges in the development of biohybrid micro/nanorobots include ensuring biocompatibility, addressing manufacturing scalability, enhancing navigation and localization capabilities, maintaining stability in dynamic biological environments, navigating regulatory hurdles, and successfully translating these innovative technologies into clinical applications. Herein, the recent advancements, challenges, and future perspectives related to the biomedical applications of biohybrid micro/nanorobots are described. Indeed, this review sheds light on the cutting‐edge developments in this field, providing researchers with an updated overview of the current potential of biohybrid micro/nanorobots in the realm of biomedical applications, and offering insights into their practical applications. Furthermore, it delves into recent advancements in the field of biohybrid micro/nanorobotics, providing a comprehensive analysis of the current state‐of‐the‐art technologies and their future applications in the biomedical field.

## Graphical abstract

This review is about biohybrid micro/nanorobots, a class of micro/nanorobotics composed of a biological part and an artificial sector. It also explores recent advancements in biomedical applications of biohybrid micro/nanorobots by focusing on their potential usage in targeted drug delivery, tissue engineering, cancer therapy, and imaging‐guided therapy.

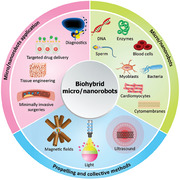

## 1 Introduction

Micro/nanorobots (MNRs) are small‐scale systems that can transform different types of energy into efficient movement for specific tasks. They have the capability to sense their surroundings, making independent decisions, and acting based on those decisions. They are also known by more specific terms like micro/nanomachine, Micro/Nanomotor, and Micro swimmers, reflecting different stages of research.^[^ ^1^ ^]^ These micro/nanorobots have made significant advances from the microscale to the nanoscale and could be fabricated using various top‐down and bottom‐up methods. They can also be fabricated in a variety of structures, including spirals, tubes, rods, needles, Janus structures, and peanut shapes. The methods for controlling these micro/nanorobots are constantly being refined, alongside continuous improvements in their motion capabilities, such as evolving from 2D to 3D motion, from simple trajectory following to obstacle avoidance, and from individual motion to coordinated cluster behavior.^[^ ^2^ ^]^

Biohybrid micro/nanorobots are an innovative class of MNRs that are designed by combining biological components with synthetic materials to create versatile and functional devices, offering new possibilities for different biomedical applications from diagnostics and targeted drug delivery to tissue engineering and minimally invasive surgeries.^[^ ^3^ ^]^ These robotic systems are fabricated using a diverse range of biological blueprints, such as DNA, enzymes, cytomembranes, blood cells, sperm, bacteria, myoblasts, and cardiomyocytes. For instance, bacterial biohybrid nanorobots use bacterial flagella to produce a corkscrew motion, and their movement can be directed using an external magnetic field. Eukaryotic cell‐based micro/nanorobots offer the possibility of using a patient's own cells to create and customize the micro/nanorobots, which enhances biocompatibility and makes their clearance from the body much easier. In this context, it refers to red blood cells (RBCs) with good flexibility and extended circulation time.^[^ ^4^ ^]^

Biohybrid micro/nanorobots offer several advantages over conventional MNRs, among the most important of them is their enhanced biocompatibility, which allows them to adapt more effectively to complex microenvironment of the body. Indeed, the use of biological components such as cells and their different organelles, proteins, or enzymes in the structure of these types of robots enhances their biocompatibility and prevents the activation of immune responses. Another factor that could help in increasing the biocompatibility feature of these robots is powering them with biocompatible chemical fuels (like glucose, urea, water, biological acids, etc.) or the use of different types of external fields (such as magnetic field, light, and ultrasound) for their movement. Additionally, it is revealed that biohybrid motors can achieve higher energy conversion efficiency compared to current artificial motors.^[^ ^5^ ^]^ Despite notable advancements in this field, significant obstacles remain, particularly in the areas of manufacturing techniques for large‐scale production and ensuring widespread clinical acceptance.

As mentioned above, researchers have been exploring the use of physical forces like magnetic fields, light, and ultrasound to collectively maneuver and guide these robotic agents.^[^ ^6^ ^]^ For instance, the appeal of magnetic actuation‐based micro/nanorobots lies in their ability to operate with fewer limitations, be remotely controlled, exhibit non‐invasive characteristics, and possess high tissue‐penetrating abilities, which has garnered significant attention in the scientific community.^[^ ^6^, ^7^ ^]^ Real‐time observation and monitoring are essential for the successful integration of biohybrid micro/nanorobots into clinical practice. Ultimately, these innovative creations hold the promise of advancing healthcare by offering precise drug dispensing systems, cutting‐edge surgical implements, and advanced diagnostic tools.^[^ ^4^, ^5^ ^]^

One of the key advantages of biohybrid micro/nanorobots is their ability to leverage the unique properties of biological components. By incorporating biological elements such as cells, enzymes, or biomolecules, these microrobots can perform complex tasks with high precision and efficiency.^[^ ^8^ ^]^ For instance, they can be engineered to target specific cells or tissues, deliver therapeutic agents directly to disease sites, or even perform localized surgeries.^[^ ^9^ ^]^ In the field of diagnostics, biohybrid micro/nanorobots offer exciting prospects. They can be designed to detect and analyze biomarkers, pathogens, or abnormal cellular activities, providing valuable insights for early disease detection and monitoring. These microrobots can navigate through body fluids or tissues, reach inaccessible areas, and enable more accurate and efficient diagnostic procedures.^[^ ^10^ ^]^ Moreover, biohybrid micro/nanorobots have the potential to improve targeted drug delivery via attaching to the specific targeting ligands or antibodies that recognize and bind to specific biomarkers present at the target site. This allows for highly specific drug delivery, ensuring that the therapeutic payload is delivered precisely to the intended location. By minimizing off‐target effects, biohybrid robots can potentially reduce the required drug dosage and minimize systemic toxicity.^[^ ^11^ ^]^

In the field of tissue engineering, biohybrid micro/nanorobots offer new possibilities for constructing functional tissues and organs. They can assist in the precise placement of cells or biomaterials, aiding in the development of complex tissue structures. In addition, these microrobots can provide mechanical stimulation or guidance cues to promote tissue regeneration and repair. These robots can be employed to manipulate and assemble cells, scaffolds, and biomaterials with high precision. They can aid in the fabrication of complex tissue structures, promote cell growth and differentiation, and facilitate the integration of engineered tissues with the host body.^[^ ^12^ ^]^

The realm of biohybrid micro/nanorobots holds immense promise for generating in vitro functional systems that cover a group of applications such as walking, swimming, gripping, and pumping.^[^ ^9^, ^13^ ^]^ Bio‐inspiration stands out as the primary method driving the advancement and utilization of biohybrid micro/nanorobots. Utilizing engineering strategies, a bottom‐up approach has been employed to design the structure, starting from individual cells, transitioning to bio‐actuators, and culminating in a fully functional biohybrid system. Recent progress in materials, 3D printing, and microfabrication opens new avenues to fabricate more sophisticated structures by merging biological muscles (incorporating cardiomyocytes, insect dorsal vessel tissues, skeletal muscle cells, and neuromuscular tissues) with inanimate materials. Despite displaying remarkable performance characteristics, biohybrid micro/nanorobots encounter challenges in accurately replicating multifaceted functions. Ensuring biocompatibility, long‐term stability, and effective control of these robots within the body are areas of active research.^[^ ^14^ ^]^ In addition, ethical considerations and regulatory frameworks need to be addressed to ensure their safe and responsible use in clinical settings.^[^ ^9^, ^15^ ^]^

Miniaturizing robots to operate at the micro‐ and nanoscales presents a significant challenge when it comes to locomotion.^[^ ^16^ ^]^ As the size of these machines decreases, they encounter obstacles due to the low Reynolds number (a dimensionless quantity used in fluid mechanics to predict the flow patterns in different fluid flow situations) environment and Brownian motion (a fundamental physical phenomenon that illustrates the random behavior of particles in fluids due to molecular collisions, reflecting the underlying kinetic nature of matter), which impede their movement. Indeed, smaller robots, particularly at the nanoscale, operate in low Reynolds number environments where viscous forces dominate over inertial forces, resulting in a more predictable, laminar flow. In contrast, microbots, being relatively larger, may operate in environments with higher Reynolds numbers, where the flow can become more complex and potentially turbulent. Additionally, nanoscale bots are heavily influenced by Brownian motion due to their small mass and the significant impact of molecular collisions, leading to more random and jittery movement. Conversely, larger microbots exhibit less sensitivity to Brownian motion, as their greater inertia dampens the effect of random molecular impacts, leading to more stable trajectories.^[^ ^17^ ^]^

To tackle this issue, designing an efficient nano/microscale machine necessitates a swimming strategy that can function within these constraints of low Reynolds numbers while employing a navigation strategy to counter the effects of Brownian motion. Conventional power supply components and batteries are impractical at such minuscule scales. Hence, innovative bioinspired design principles are imperative to address the demanding requirements for power supply and locomotion. Over the past decade, various types of micro/nanorobots have been created based on different actuation principles such as magnetic, electric, acoustic, and light fields as well as chemical and biological methods.^[^ ^18^ ^]^

Chemically powered motors propel themselves through aqueous solutions by inducing surface reactions to create local gradients of concentration, electrical potential, or gas bubbles. Magnetic swimmers mimic the movements of natural swimming microorganisms by employing magnetic actuation with helical or flexible flagella. Acoustic nanomotors utilize asymmetric steady streaming to generate propulsion speed in specific directions, while other devices harness optical, thermal, and electrical energies for distinctive locomotion principles. Moreover, the integration of synthetic micro/nanodevices with motile organisms has given rise to biologically powered hybrid nanorobots, showcasing a diverse array of propulsion methods and leading to the development of various micro/nanorobotic prototypes.^[^ ^16^ ^]^

This perspective on the biomedical applications of biohybrid micro/nanorobots stands out from other reviews on biomedical applications of micro/nanorobots due to its specialized focus on the integration of biological components with synthetic materials. Unlike generic reviews that encompass all types of micro/nanorobots, this review delves specifically into biohybrid systems, exploring their unique advantages and challenges. With the integration of biological organisms, such as cells or tissues, with artificial components, this perspective offers a concise description about how biohybrid micro/nanorobots can revolutionize biomedical applications. Furthermore, it delves into recent advancements in the field of biohybrid micro/nanorobotics, providing a comprehensive analysis of the current state‐of‐the‐art technologies and their implications for the biomedical domain. Thus, this perspective adds value by offering a specialized examination of biohybrid micro/nanorobots and their specific relevance in biomedical settings, thereby serving as a valuable resource for researchers seeking in‐depth insights into this emerging field of study.

## 2 Biomedical Applications of Biohybrid Micro/Nanorobots

Micro/nanorobots are a class of advanced materials considered among the most prominent topics in technology, with the ability to move autonomously in the physiological environment and so they could be candidates for different biomedical applications;^[^ ^19^ ^]^ however, their probable toxicity in the biological systems has restricted in vivo applications of micro/nanorobots. The integration of microorganisms and biological cells with these micro/nanorobots leads to improve their biocompatibility, overcome their systemic toxicity, and inhibit their immune response resulting in better performance compared to traditional methods. Indeed, the autonomous ability of these robots has made them the focus of several research in different sectors of biomedicine, offering unprecedented possibilities for targeted drug delivery, diagnostics, and minimally invasive surgeries.^[^ ^5b^ ^]^

They could move easily through the bloodstream and deliver therapeutic compounds directly to their targeted site, while reducing side‐effects and improving therapeutic performance. They can also be used for the diagnosis via providing highly sensitive imaging and detecting method at the molecular level. In the context of minimally invasive surgeries, nano/microrobots have the potential to perform intricate tasks, such as tissue repair or removal of abnormalities, with precision that surpasses conventional methods.^[^ ^20^ ^]^

### 2.1 Targeted Drug Delivery

Insufficient delivery of therapeutic compounds to their target cells is considered as the main reason of unsuccessful treatment of hard‐to‐treat disease like different types of cancer, neurodegenerative disease, etc. Indeed, for the precise administration of therapeutic medications to specific areas of the body, it is crucial to introduce new delivery vehicles that possess controlled navigation, propelling force, cargo‐towing or release, and tissue penetration capabilities. Advances in nanotechnology and microfabrication techniques have paved the way for the development of tiny robotic surgeons, known as micro/nanorobots, that hold the potential to access to the challenging and distant regions within the body, facilitating medical interventions. They can encapsulate therapeutic compounds, enhance their rapid accumulation in diseased tissue, release them in a controllable manner, and enhance their therapeutic performance, while reducing dosage and their probable side‐effects.^[^ ^21^ ^]^ The conjugation of biological cells with artificial components, which have the capability of converting chemical energy into mechanical work or other cellular functionalities, or motile microorganisms, like bacteria, sperm, and contractile and immune cells, are identified as the potential engines for propelling biohybrid microrobots, especially for drug delivery applications.^[^ ^22^ ^]^

In one study, *Thalassiosira weissflogii* frustules (TWFs) diatoms, as a natural porous silica structure, were functionalized with magnetic nanoparticles using electrostatic interactions. The porous structure of these diatoms provided an ideal surface for loading high amounts of drug (doxorubicin (DOX)). These microrobots exhibited movement in the presence of external magnetic field that provided the capability of targeting toward the diseased cells. Based on the frequency of this magnetic field, these microrobots showed two different movement patterns; their speed increased in the presence of higher frequency that led to faster delivery to their targeted site, while they showed slow movement in response to using lower frequency that enabled them to pass channels that have smaller widths than their length. Drug release from this microrobot showed a burst release at first 8 h followed by a sustainable pattern. Besides, the amount of released drug was higher in acidic pH compared with the neutral one that confirmed pH‐responsivity of this system. Treating cancer cells with this microrobot led to a significant toxicity effect via affecting cellular membrane.^[^ ^23^ ^]^

**Figure 1:**
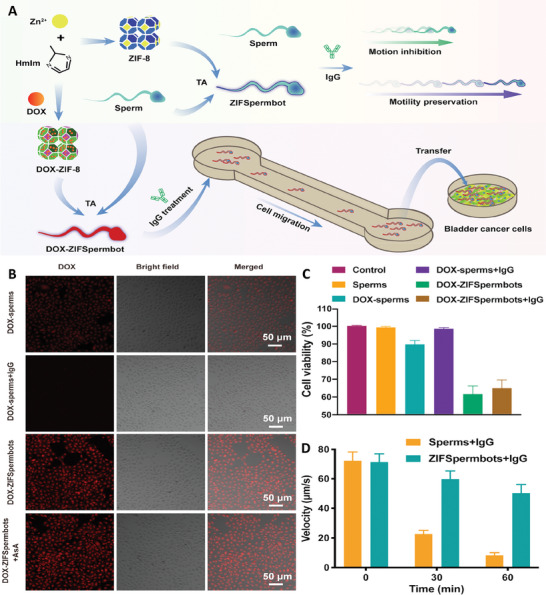
A) Schematic image related to the fabrication and application of ZIFSpermbots as drug delivery system for cancer therapy. Fluorescence images B) and cell viability test C) of 5637 bladder cancer cells exposed with different treatments. D) Effect of utilizing ZIF on velocity of microrobots contained IgG. Reproduced with permission.^[^ ^24^ ^]^ Copyright 2021, American Chemical Society.

Functionalizing sperms with zeolitic imidazolate framework‐8 nanoparticles (ZIF‐8 NPs) (ZIFSpermbots) in the presence of tannic acid (TA) led to the fabrication of a type of biohybrid microrobot for drug delivery. Presence of ZIF‐8 NPs, with their porous structure, provided a widespread surface for loading therapeutic compounds, protected sperms against antisperm antibodies (AsA), and acted as an antioxidant compound via releasing Zn^2+^ ions, which protected the movement of sperms in harsh environment. Moreover, this antioxidant property also protected spermatozoa from the reactive oxygen species produced inside them due to the peroxidation of their membrane polyunsaturated fatty acids. DOX loaded ZIFSpermbots (in the presence and absence of AsA) exhibited significant anticancer activity against 5637 bladder cancer cell line that confirmed the effectiveness of this microrobot (**Figure** **1**).^[^ ^24^ ^]^

Immune cells are another type of material that could be used for the fabrication of biohybrid microrobots. For instance, a neutrophil‐based microrobot was fabricated, as a drug carrier by producing paclitaxel (PTX)‐loaded magnetic nanogels coated with membrane of *Escherichia coli* (*E. coli*) that were then phagocytosed by the natural neutrophils. It was a dual responsive carrier exhibited the capability of magnetic movement within blood vessels in the presence of a rotating magnetic field (RMF) and chemotaxis in response to a gradient of inflammatory factors that enabled it to actively navigate the blood‐brain barrier (BBB) to target malignant gliomas. Utilizing the outer membrane of *E. coli* not only prevented drug leakage within the neutrophils but also significantly improved their encapsulation by neutrophils within 30 min. Encapsulation of bacterial membrane coated PTX‐loaded magnetic nanogels inside neutrophiles did not affect the morphology of neutrorobots and the viability of neutrophils. Drug release from this robot was evaluated under different conditions including blood stream and in the presence of magnetic field and inflammatory factors gradient. Indeed, the inflammatory gradient led to the chemotactic motion, showing very low drug release in low inflammatory condition and a significant release in the presence of inflammation, confirming the role of inflammatory conditions in releasing therapeutic compound from the neutrorobots. In the presence of external magnetic field, the neutrorobots were delivered to the BBB and then actively crossed it in response to the presence of gradient of inflammatory compounds, as confirmed by the Transwell system. This resulted in significant toxicity effect during in vitro and in vivo test that was related to the accumulation of drug loaded neutrorobots in the disease site, while no toxicity effect was observed in vital organs.^[^ ^25^ ^]^

Covalent conjugation of *Chlamydomonas reinhardtii* microalgae with vancomycin, using disulfide bond, was used in a study to fabricate a smart thiol responsive antibacterial biohybrid microrobot. In the presence of reducing agents and at 37 °C drug release from this system was enhanced, with nearly 76% of loaded drug was released within 6 h. The antibacterial activity was assessed against *Bacillus subtilis* (*B. subtilis*) and *Staphylococcus aureus* (*S. aureus*) (MRSA) in the presence of reducing agent that the results showed the effectiveness of the microrobot in inhibiting growth of bacteria within just 14 h.^[^ ^26^ ^]^

*Chlorella* (*Ch*.) cells functionalized with Fe_3_O_4_ nanoparticles were used for the targeted delivery of DOX molecules. Utilizing dynamic magnetic self‐assembly led to the reconfiguration of monomers into the biohybrid microrobot multimers (BMMs) that were changed to branch‐like structure under the in‐plane rotating magnetic fields. Indeed, they exhibited reversible assemble ability that enabled them to intelligently control their swarm in different environments. They also showed pH responsive drug release pattern so that the amounts of released drug were enhanced by decreasing pH. Results of targeting test confirmed the ability of the robots to actively reach their target site in an actively manner and in the presence of external magnetic field. The fabricated microrobot had no toxicity effect on cancer cells while drug loaded one significantly decreased the viability of cells even in low concentration of DOX (20 µg ml^−1^), which was related to the effect of microrobot on the membrane of the cells that led to induce apoptosis to the cells.^[^ ^27^ ^]^

**Figure 2:**
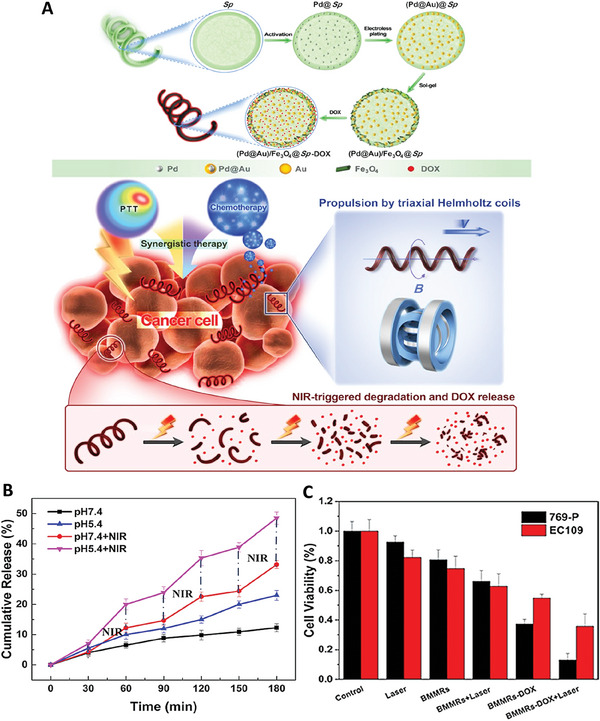
A) Schematic illustration of fabrication and application of (Pd@Au)/Fe_3_O_4_@*Sp*.‐DOX microrobots for drug delivery to cancer cells. B) Drug release from the microrobots incubated under different conditions. C) Results of cell viability test exposed with different treatments. Reproduced with permission.^[^ ^28^ ^]^ Copyright 2019, American Chemical Society.

Microrobots could be designed so that they could exhibit combination therapy effect. For instance, *Spirulina*, a type of blue‐green algae with 3D helical structure, was induced to fabricate Pd@Au nanoparticles using electroless deposition method. The (Pd@Au)@Sp microrobots were then functionalized with iron oxide nanoparticles and then loaded with DOX, as anticancer drug. In this system, the presence of Pd@Au nanoparticles provided the capability of photothermal effect for this microrobot in response to near‐infrared (NIR) irradiation. On the other hand, presence of iron oxide nanoparticles provided the ability of active targeting to the cancer cells in the presence of an external magnetic field. It was a smart robot that exhibited pH and laser responsive drug release pattern, so that the amounts of released drug were increased by decreasing pH or utilizing laser irradiation. The combination application of laser and chemotherapy led to a significant anticancer activity resulting from the chemo‐photothermal therapy effect of the fabricated microrobots (**Figure** **2**).^[^ ^28^ ^]^

**Figure 3:**
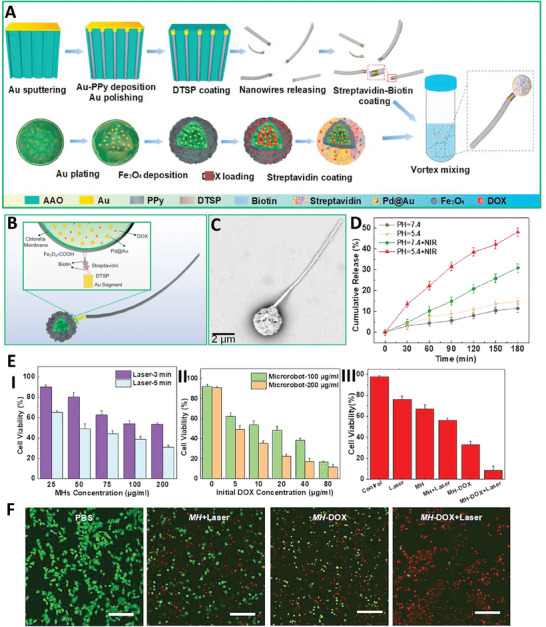
A) Schematic illustration of BFSMs microrobot fabrication. Schematic B) and scanning electron microscopy (SEM) (C) images of BFSMs microrobot. D) Drug release curve of DOX in the presence of different pH and NIR irradiation. E) Cell viability results of HeLa cells in the presence of different concentrations of magnetic spherical heads (I), DOX (II), and different treatment (III). F) Results of Live/dead cell test in the presence of different treatment (Scale bar: 200 µm). Reproduced with permission.^[^ ^29^ ^]^ Copyright 2024, American Chemical Society.

Inspired by sperms, a flexible biohybrid microbot (BFSMs) was produced, composed of *Chlorella* (*Ch*.) cells as a template which were completely covered with multi‐shells, including Pd@Au core‐shell nanoparticles, Fe_3_O_4_ nanoparticles, and streptavidin. The head part was loaded with DOX as an anticancer drug. Nanowires of Au/polypyrrole (PPy) were synthesized via electrochemical method and used as the flexible tails, which were attached to the head part via biotin‐streptavidin interaction. Utilizing an external magnetic field led to the flexible movement of the fabricated microbot in the direction of this magnetic field without exhibiting U‐turn. Presence of high numbers of nanochannels in the structure of head part provided a wide space for loading large amounts of DOX inside the channels and also on the surface of magnetic spherical heads (MH). Interestingly, the presence of Pd@Au nanoparticles in the structure of this microbot provided the capability for photothermal therapy, such that utilizing 808 nm laser irradiation for 10 min led to increasing the temperature to ≈44 °C. The fabricated formulation showed concentration‐dependent and power‐dependent photothermal effect. Besides, it showed pH‐ and temperature‐dependent drug release pattern so that it could release near 50% of DOX within 180 min in acidic pH and under NIR irradiation, while less than 10% release occurred at normal pH. It also showed concentration‐dependent and laser exposure time‐dependent cytotoxicity against cancer cells, with higher concentrations of nanoparticles and longer laser irradiation times resulting in greater toxicity. Utilizing 200 µg ml^−1^ of microbot with 40 µg ml^−1^ DOX and 5 min laser irradiation led to more than 90% toxicity against HeLa cells, which was significantly higher than other samples and was related to the combination effects of chemo‐ and photothermal therapies (**Figure** **3**).^[^ ^29^ ^]^

### 2.2 Tissue Regeneration and Cancer Therapy

Tissue regeneration is a dynamic and intricate process within the field of regenerative medicine that occurs via a series of cellular and molecular events with the aim of restoring the structure and functions of tissues. The advancements in tissue regeneration hold immense promise for addressing various medical conditions, ranging from traumatic injuries to degenerative diseases, with the ultimate aim of improving patient outcomes and quality of life. However, it suffers from some obstacles including invasive surgical techniques, limited targeting precision, and side effects such as local tissue trauma. These issues can impact the effectiveness of treatment, impede postoperative recovery, and result in discomfort. To attain accurate therapeutic interventions with minimal invasiveness and without undesirable side effects, researchers have suggested utilization of miniaturized synthetic micro/nanomotors. These devices are designed to deliver stem cells precisely to the damaged tissues or serve as a minimally invasive platform for converting external energy into regenerative signals. In this context, nano/microrobots exhibit unique characteristics such as their diminutive size, high precision, and efficiency, that make them appropriate for tissue regeneration applications.^[^ ^30^ ^]^ For instance, decellularized cartilage extracellular matrix (ECM) functionalized with Fe_3_O_4_ nanoparticles (via dip coating) was used as scaffold for the delivery of human bone marrow mesenchymal stem cells (MSCs) for osteoarthritis (OA) treatment. The porous structure of scaffold provided a good space for seeding cells. After reaching their targeted site, MSCs were released from the scaffold within 12 h and started to proliferation. Moreover, 20 days after cultivation, the scaffold was degraded. Results of in vivo tests confirmed the effectiveness of this formulation in recovery of knee joint function within 3 weeks, highlighting the importance of presence of both functional cells and microenvironments for this aim.^[^ ^31^ ^]^

*Chrysanthemum* pollen‐derived biohybrid magnetic microrobots (CDBMRs) were fabricated via coating iron nanoparticles with a *Chrysanthemum* pollen shell that has a porous structure. They showed targeting ability in the presence of an external magnetic field with magnetic velocity and input frequency dependency, such that by increasing the magnetic frequency until critical frequency velocity increased as well, while passing this critical point led to a decrease in velocity. This magnetic property provided the capability of delivering this microrobot to its target site using an external magnetic field. At the target location, microrobots were attached to cells and penetrated into them via time‐varying magnetic field. Presence of burr‐like micro spikes on the surface of this microrobot induced cell apoptosis due to cell perforation. Moreover, its hollow structure provided a high capacity for loading therapeutic compounds and released them at the target site. Besides, its porous and burr‐like microstructure made it suitable for the attachment and delivery of cells. It was shown that the external magnetic field could induce osteogenic differentiation and improve bone growth via electrically stimulating the cells that confirmed its tissue regeneration ability.^[^ ^32^ ^]^

**Figure 4:**
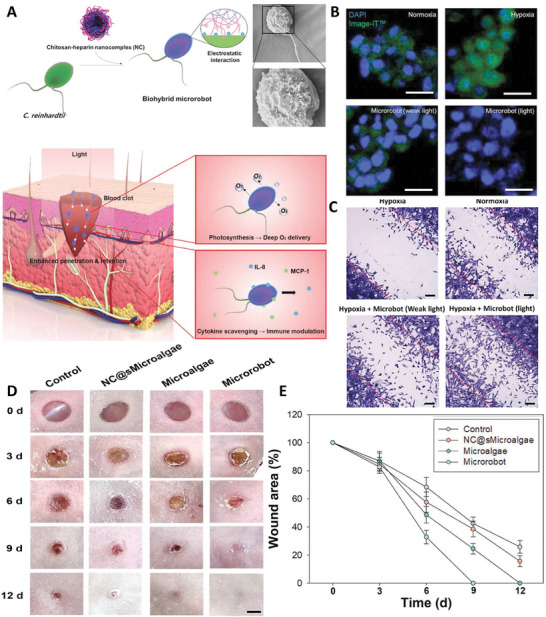
A) Scheme of chitosan‐heparin nanocomplex coated microalgae used for wound healing application. Oxygen production (Scale bar = 50 µm) B) and cellular migration (Scale bar = 10 µm) C) of NIH 3T3 fibroblast cells exposed with different treatments. Results of wound healing D) and percentage of wound area E) in diabetes mice exposed with different treatments. Reproduced with permission.^[^ ^33^ ^]^ Copyright 2022, Wiley.

A biohybrid microrobot was fabricated via incorporating chitosan‐heparin nanocomplex (NC) into the *Chlamydomonas reinhardtii (C. reinhardtii)* microalgae for accelerating wound healing. It had the ability to penetrate through the blood clots and produce oxygen in the microenvironment of diabetic wound via photosynthesis that reduced hypoxic conditions of wound. Besides, presence of polyanion groups of heparins provided the capability to interact with positively charged groups of cytokines and scavenge them, that led to the modulating the inflammatory conditions of wound. Presence of these microrobots in the microenvironment of fibroblast cells under hypoxic condition and light induced the proliferation and migration of these cells (compared to control test) that confirmed the effectiveness of these microrobots in production of oxygen. Interestingly, results of in vivo test showed that after healing of wound no microrobot remained in the wound site, which confirmed their clearance after healing process. These microrobots not only enhanced fibroblast proliferation and migration, but also improved collagen deposition, neo‐angiogenesis, and tissue regeneration (**Figure** **4**).^[^ ^33^ ^]^

**Figure 5:**
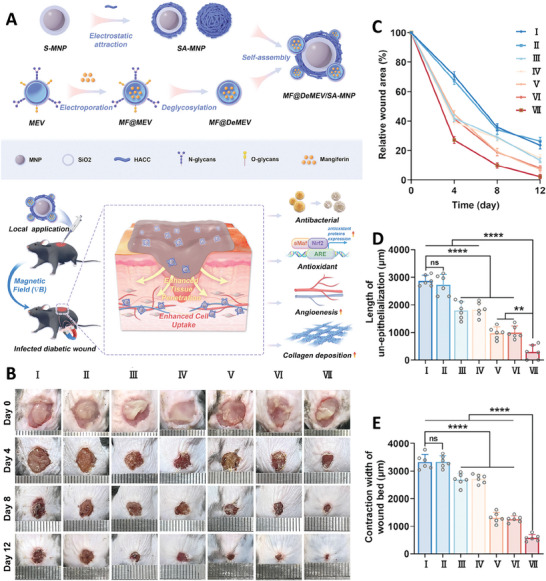
A) Schematic image of fabrication and wound healing application of MF@DeMEV/SA‐MNP nanorobot. Results of wound healing effect B), wound area curve C), length of un‐epithelialization curve D), and change in wound bed's width E) of infected wounds exposed with different samples (including I: Control, II: Free MF, III: SA‐MNP+∇B, IV: DeMEV/SA‐MNP+∇B, V: MF@MEV/SA‐MNP+∇B, VI: MF@DeMEV/SA‐MNP, and VII: MF@DeMEV/SA‐MNP+∇B). Reproduced under the terms of the CC‐BY license.^[^ ^34^ ^]^ Copyright 2024, Yan et al., published by Wiley‐VCH GmbH.

To overcome the limitations related to the treatment of diabetic wounds, a type of biohybrid nanorobot (MF@DeMEV/SA‐MNP) was fabricated composed of an artificial core‐shell nanoparticle (the Fe_3_O_4_ magnetic nanoparticle covered with SiO_2_ and functionalized with antibacterial 2‐hydroxypropyltrimethyl ammonium chloride chitosan (HACC)) that was functionalized with the biological part (contained mangiferin (MF) loaded inside the milk‐derived extracellular vesicles (MEVs)). In this system, the artificial part, with antibacterial feature, was responsible for targeting the nanorobots to the damaged part and enhancing their cell penetration, while the biological part enhanced cellular penetration of the particles and provided the antioxidant feature for the robots. According to the results of cytocompatibility and hemocompatibility, the fabricated nanorobots were biocompatible at concentrations lower than 100 µg ml^−1^. The fabricated nanorobots utilized macropinocytosis and caveolae‐dependent endocytosis pathways to penetrate into the endothelial cells and macropinocytosis and clathrin‐mediated endocytosis pathways to enter fibroblast cells. Moreover, applying a gradient magnetic field led to the fabrication of a needle‐like structure by the robots for penetrating into the deeper parts. By exhibiting antioxidant activity, the fabricated nanorobot could activate the proliferation and migration capability of endothelial cells and fibroblasts and angiogenesis in endothelial cells. The antibacterial activity of the nanorobots was evaluated against *Staphylococcus aureus* and *E. coli* that confirmed high antibacterial activity of nanorobots which was improved in the presence of magnetic field. Exposing infected wound of diabetic mice with the fabricated nanorobots led to healing wound for 12 days compared to other samples. This therapeutic performance was better in samples exposed with magnetic field so that it showed more regular collagen deposition, high pro‐angiogenic effect, and excellent antibacterial activity (**Figure** **5**).^[^ ^34^ ^]^

One of the interesting methods introduced in recent years to address the limitations of current cancer treatment approaches is the development of controllable drug delivery micro/nanorobots. These robots aim to precisely targeting the affected areas using active targeting methods, with minimal invasiveness. The key benefits of these robots include accurate lesion site targeting and reduced negative impact on normal cells.^[^ ^35^ ^]^ By the improvement of technology, various robotic paradigms have been introduced, and one of the most interesting types is biohybrid robots, which involves integrating carefully designed artificial structures with living biosystems. This approach holds promise for overcoming barriers in cancer treatment, showcasing the dynamic intersection between engineered technology and living biological systems.^[^ ^36^ ^]^

**Figure 6:**
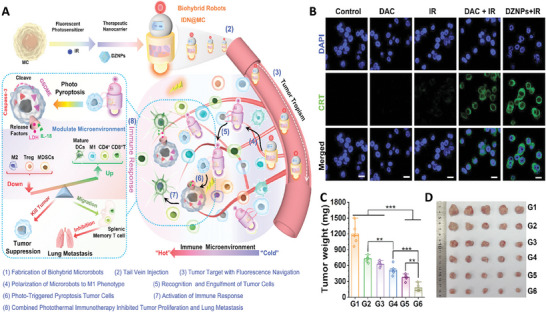
A) Schematic of macrophages‐based biohybrid microrobots used for cancer therapy. B) Fluorescence images of 4T1 cells exposed with Calreticulin (CRT) and different microrobots. Effect of different treatment on tumor weight C) and tumor size D). Reproduced with permission.^[^ ^37^ ^]^ Copyright 2023, Wiley.

In a study, indocyanine green, a photosensitizer, and decitabine (DAC) loaded ZIF‐8, a therapeutic compound, were loaded inside RAW 264.7 macrophages to fabricate a new type of biohybrid microrobot with the ability of cancer treatment using photothermal and immunotherapy. Loading DAC inside the smart MOF not only improved the bioavailability of this compound (via protecting it against cytidine deaminase and hydrolytic cleavage), but also provided the capability of releasing drug in a controllable manner and in response to changing pH in the microenvironment. In this study, DAC was used to induce pyroptosis in the cancer cells and thereby enhanced the sensitivity of these cells to the treatment. These ZIF nanoparticles showed the capability of locating in the nuclei zone through breaking the nuclei membrane. Utilizing 2 min NIR irradiation rapidly increased the temperature of indocyanine green microrobot to ≈45 °C that confirmed the photothermal ability of the microrobot. Sustained degradation of drug loaded ZIF led to the release of drug and Zn^2+^ ions that affect macrophages and changed their genetic to fabricate M1‐type macrophage. Co‐culturing of microrobots with 5(6)‐carboxyfluorescein diacetate N‐succinimidyl ester (CFSE)‐labeled 4T1 cells revealed the attachment of microrobots to the cells within 30 min, while most of them were phagocytosed by the microrobots after 12 h of imaging. Utilizing NIR irradiation led to release of therapeutic agents to the microenvironment of tumor cells which could then diffuse into the cells and induce photothermal effect followed by the activation of T‐cells that killed cancer cells. Indeed, the combination use of PTT and chemotherapy led to synergistically induced pyroptosis in cancer cells, exposing calreticulin on the surface of cancer cells and induced maturation of dendritic cells and production of different immunocytokines which led to the activation of immune responses. The fabricated microrobot showed the capability of escaping from the reticuloendothelial system, reaching tumor tissue in a controllable manner, and effectively reducing the tumor size in real samples (**Figure** **6**).^[^ ^37^ ^]^

*E. coli* Nissle 1917 (EcN) was applied in a study to fabricate microrobot with the ability of active drug delivery to the deeper parts of tumor tissue. In this study, magnetic nanoparticles were synthesized and functionalized on the surface of genetically modified EcN bacteria that had the capability for imaging‐guided magnetothermal treatment (due to the addition of NDH‐2 or mCherry/NDH‐2 gene sequences as well as a thermally sensitive promoter (TcI) via using pBV220 expression vectors). Utilizing 6 min of alternating magnetic field (AMF) led to an increase in the temperature of aqueous solution of microrobots to more than 42 °C, which then activated their magnetothermal bio‐switch. Results of live/dead cells on bacteria showed that the addition of nanoparticles and gene didn't affect the proliferation and viability of bacteria that confirmed the compatibility of bacteria with the added genes and nanoparticles. These microrobots had the capability to attach to the cell surface via their flagella and penetrating into the cells. After penetrating into the cells and in the presence of AMF, elevated level of reactive oxygen species (ROSs) was fabricated inside the cells that induced cellular apoptosis. On the other hand, the acidic condition of cancer cells led to the release of Fe ions from the microrobots that activated the Fenton reactions and enhanced toxicity effect via inducing peroxidation of lipids. They could accumulate in deeper parts of tumor site due to the presence of hypoxic condition of tumor microenvironment as well as the outside magnetic field. Applying AMF also led to increasing the temperature up to 45 °C in 10 min. Results of in vivo tests confirmed the effectiveness of this microrobot in treating cancer tissues without affecting other organs and also were eliminated from the targeted site after treatment process.^[^ ^38^ ^]^

**Figure 7:**
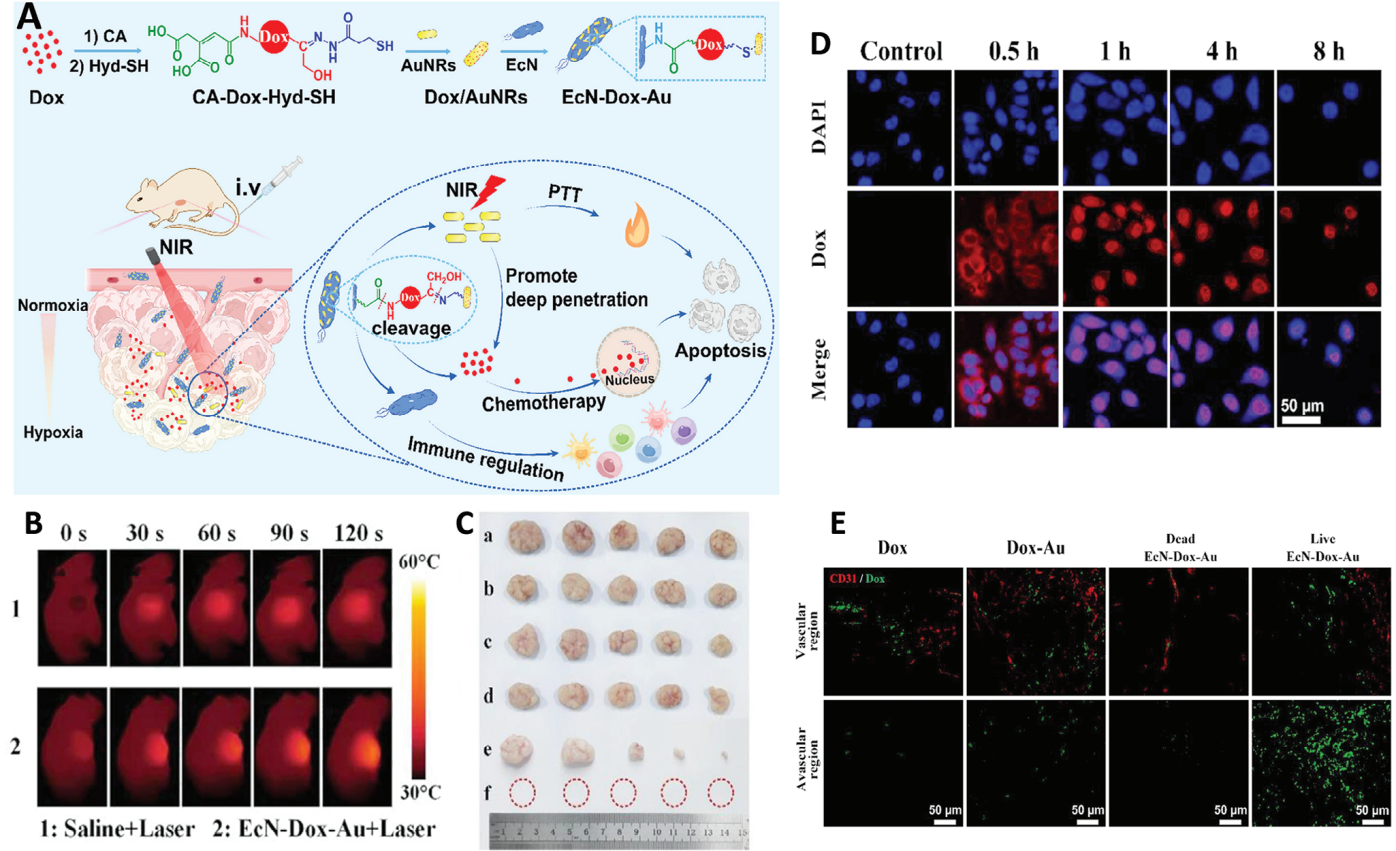
A) Schematic image related to the fabrication and application of EcN‐Dox‐Au microrobots as multimodal compound for breast cancer therapy. B) Thermographs of tumor‐bearing mice treated with microrobot and laser (2 min) during the time. C) Image of tumor treated with different formulation a) saline, b) Dox, c) EcN, d) EcN‐Dox‐Au, e) AuNRs + laser, and f) EcN‐Dox‐Au + laser). D) Localization of microrobots inside the cells during the time. E) Distribution of Dox, CA‐Dox‐Hyd‐SH/AuNRs complexes (Dox‐Au), and live and dead EcN‐Dox‐Au conjugates in avascular and vascular region of tumor. Reproduced with permission.^[^ ^39^ ^]^ Copyright 2023, Elsevier.

In another study, a pH responsive nanotheranostic platform was fabricated first via conjugating DOX functionalized with cis‐aconityl anhydride (CA) and thiol‐decorated hydrazone derivative (Hyd‐SH) with gold nanorods (AuNRs) (as a photosensitizer). Then, these nanocompounds were attached on the surface of EcN bacteria to fabricate biohybrid microrobot for the aim of cancer therapy. It showed sufficient mobility toward cancer tissue; however, its mobility was less than that of EcN. Utilizing NIR irradiation for 10 min, led to increasing the temperature in AuNRs dose dependent manner. Moreover, it showed pH‐responsive drug release so that it had just 30% release at normal pH, while exhibited 70% release at acidic pH, therefore it could have good therapeutic performance against cancer cells (due to acidic pH of their microenvironment), while had less effect on normal cells. It also had high anticancer activity, even in low concentration, which was significantly improved in the presence of NIR irradiation. These microrobots were internalized by the cells and accumulated inside the nuclei. Results of in vivo test also showed increasing the amounts of microrobots inside the tumor tissue, while decreasing their level in other organs during the time. Besides, there was a notably elevated concentration of EcN was observed in the kidneys, which was diminished by ≈100‐fold across various organs after a 7‐day period suggesting the rapid clearance of EcN from the tissues, likely attributed to the swift recognition and elimination of lipopolysaccharide sensitive to serum. They had accumulated inside the deep parts of tumors, due to their affinity to hypoxic microenvironment, and induced significant anticancer activity resulted from chemo‐photothermal therapy as well as triggering immunomodulatory effect (**Figure** **7**).^[^ ^39^ ^]^

As mentioned before, RBCs are one of the best candidates that could be used for the fabrication of biocompatible biohybrid robots that have prolonged circulation time. The RBC membrane was used in a study to fabricate magnetic navigable RBC‐mimicking (RBCM) micromotors containing indocyanine green (ICG) and RBC‐shaped hemoglobin particles loaded with Fe_3_O_4_ nanoparticles. Due to their special structure (the RBC‐shaped biconcave discoidal structure), these micromotors could freely move through the blood vessels, without activating immune response, using ultrasonic energy as their fuel. Besides, utilizing an external magnetic field enabled them to move toward their targeted tissue. The fabricated micromotor showed interesting features like antifouling ability, high cytocompatibility, very low hemolysis ability, good stability, and anti‐phagocytosis capability. At the site of cancer cells, utilizing the oxygenated micromotor and NIR light led to an extraordinary cytotoxicity effect related to the production of ROSs inside the cancer cells due to the photodynamic effect of ICG and oxygen molecules.^[^ ^40^ ^]^

**Figure 8:**
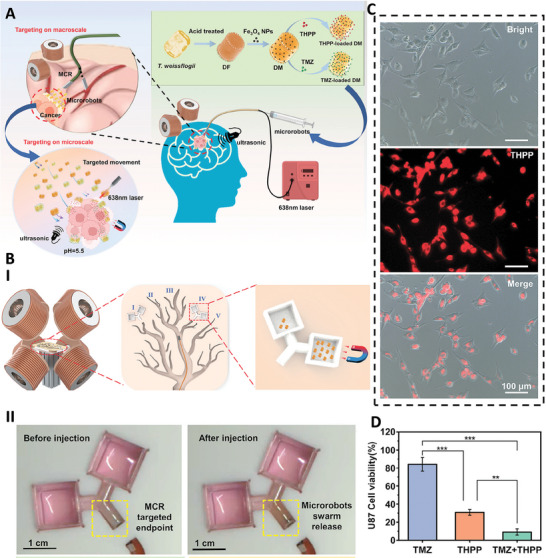
A) Schematic image of TMZ and THPP‐loaded microbots fabrication used for the treatment of glioblastoma via chemo‐photodynamic therapy. B) Utilizing MCR for cross‐scale delivery of microbots (I). Change in the color related to release of microbot swarm (II). C) Fluorescence image of U87 cells treated with THPP‐loaded microbots. D) Result of cell viability assay of U87 cells treated with TMZ‐loaded microbots, THPP‐loaded microbots, and their combination (**p < 0.01, and ***p < 0.001). Reproduced with permission.^[^ ^41^ ^]^ Copyright 2024, Wiley.

In a recent study, the *Thalassiosira weissflogii* diatom was used to fabricate magnetic microbots for the combination therapy of glioblastoma. In detail, the natural diatom frustules (DFs) were first functionalized with Fe_3_O_4_ nanoparticles, using a vacuum method, and then temozolomide (TMZ), an anticancer drug, and 5,10,15,20‐Tetrakis(4‐hydroxyphenyl)porphyrin (THPP), were loaded inside microrobots, separately. The fabricated microbots had the capability of movement toward a targeted site, in the presence of an external magnetic field, as well as specific abilities for reverse movement and swarm migration. They also showed the ability of releasing TMZ and THPP in pH‐responsive and ultrasound‐responsive manners, respectively. Five‐coil magnetic system was used to provide a rotating magnetic field for targeting the microbots toward cancerous tissue. The fabricated microbot showed controllable and flexible movements with the ability of moving against flow in the presence of magnetic field. Utilizing microbots without drug or THPP‐loaded microbots at dark conditions had no toxicity effect against cancer cells, while light irradiated THPP‐loaded microbots showed cytotoxicity results even at low concentration. Besides, applying the combination of TMZ‐loaded microbots (30 µg ml^−1^) and THPP‐loaded microbots (20 µg ml^−1^) led to a significant reduction in viability of the cancer cells (to less than 10% and 40%, respectively). In the following part, magnetic continuum robot (MCR) was used for crossing microbots along the macroscale due to their capability to bypassing intricate functional areas of the brain and accessing deeper regions during glioma treatment. Then, microbots could reach to their targeted site via cross‐scale drug delivery manner and in the presence of magnetic field. Utilizing these methods led to significant cytotoxicity effect on glioblastoma cancer cells (less than 20%), while the normal cells were viable. Therefore, the combination of MCR and magnetic based biohybrid microbots could be considered as a good method for delivery of therapeutic compounds to deep parts of brain (**Figure** **8**).^[^ ^41^ ^]^

### 2.3 Diagnostic and Imaging Techniques

Incorporation of imaging modalities, such as fluorescence or magnetic compounds, into the biohybrid microrobots introduces them as diagnostic compounds for real‐time monitoring of specific biological targets. They could also be used for the selective detection of cells, proteins, bacterial toxins, metal ions, etc. Besides, the biocompatibility of these biohybrids enhances their potential for in vivo applications.^[^ ^10^ ^]^ For instance, *Spirulina* microalgae functionalized with Fe_3_O_4_ nanoparticles were used in a study for the aim of imaging guided treatment. The fabricated microrobot showed the capability of movement within different media from DI water to urine, blood, and gastric juice. This microrobot had the capability of acting as both fluorescence compound and contrast agent for magnetic resonance imaging (MRI), which were related to the microalgae and nanoparticles, respectively. The intensity of this fluorescent property had an inverse relationship with the time of nanoparticles coating so that by increasing the time of coating, the autofluorescence ability of microalgae was decreased. Its fluorescence ability showed pH dependency, as well, so that in neutral and basic pH it had strong fluorescence ability, while in acidic solution, a weak intensity was detected. Besides, in the deeper parts of body, the MR imaging could be applied due to the weakness of fluorescence images.^[^ ^42^ ^]^

**Figure 9:**
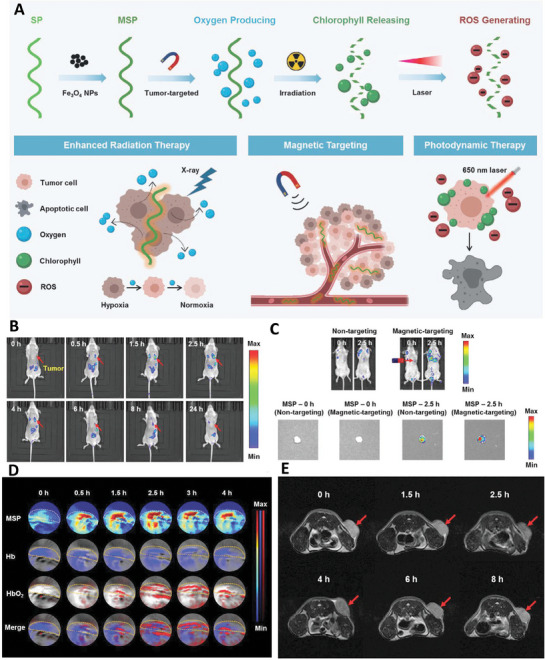
A) Schematic image related to the fabrication and application of biohybrid microrobot for imaging and treatment of tumor. In vivo fluorescence imaging B) during 2.5 h post‐injection and C) in the presence/absence of magnetic targeting. Photoacoustic image D) and T_2_ MR image E) of mice treated with magnetic *S. platensis* (MSP) hybrid microswimmers during the time. Reproduced with permission.^[^ ^43^ ^]^ Copyright 2020, Wiley.

*Spirulina platensis* (*S. platensis*) microrobots functionalized with Fe_3_O_4_ nanoparticles were fabricated in another study for cancer multimodal imaging and therapy. Presence of *S. platensis* in the structure of this microrobot provided it with the capability of producing oxygen molecules under irradiation. Presence of chlorophyll with autofluorescence ability in the structure of *S. platensis* introduced this microrobot as a cost‐effective fluorescence modality. The chlorophyll also furnished the microrobots with the ability of photoacoustic (PA) imaging. On the other hand, iron oxide nanoparticles provided the capability of T_2_‐weight MR imaging from the tissue treated with these microrobots. Utilizing out‐source magnetic field led to the accumulation of fabricated microrobots in the tumor tissue, which was confirmed by the fluorescence imaging. The intensity of PA imaging was improved by the time resulted from the accumulation of microrobots inside the tumor cells and production of oxygen molecules that increased the level of HbO_2_. This oxygen generation also led to eliminating the hypoxic conditions of tumor tissue. The fabricated microrobots showed degradation ability that confirmed their clearance from the body after finishing treatment process (**Figure** **9**).^[^ ^43^ ^]^

Chlorin e6 was loaded inside the chitosan/polydopamine functionalized magnetic (Ce6‐CS/PDA@Fe_3_O_4_) nanoparticles which were then functionalized on the surface of volvox algae to fabricate theranostic biohybrid microrobots. Presence of chlorophyll in the algae provided the capability of photoacoustic and fluorescence imaging for these microrobots. Moreover, iron oxide nanoparticles could be detected by the MRI and photothermal imaging techniques. Irradiation of these microrobots with 660 nm laser light led to the production of oxygen from one side and converting the oxygen into the singlet oxygen from the other side. Moreover, Fe_3_O_4_ nanoparticles could act as targeting agent from one side, and generate photothermal effect that killed cancer cells, from the other side. These microrobots had the ability of movement in different biological media without affecting blood cells and organs.^[^ ^44^ ^]^

**Figure 10:**
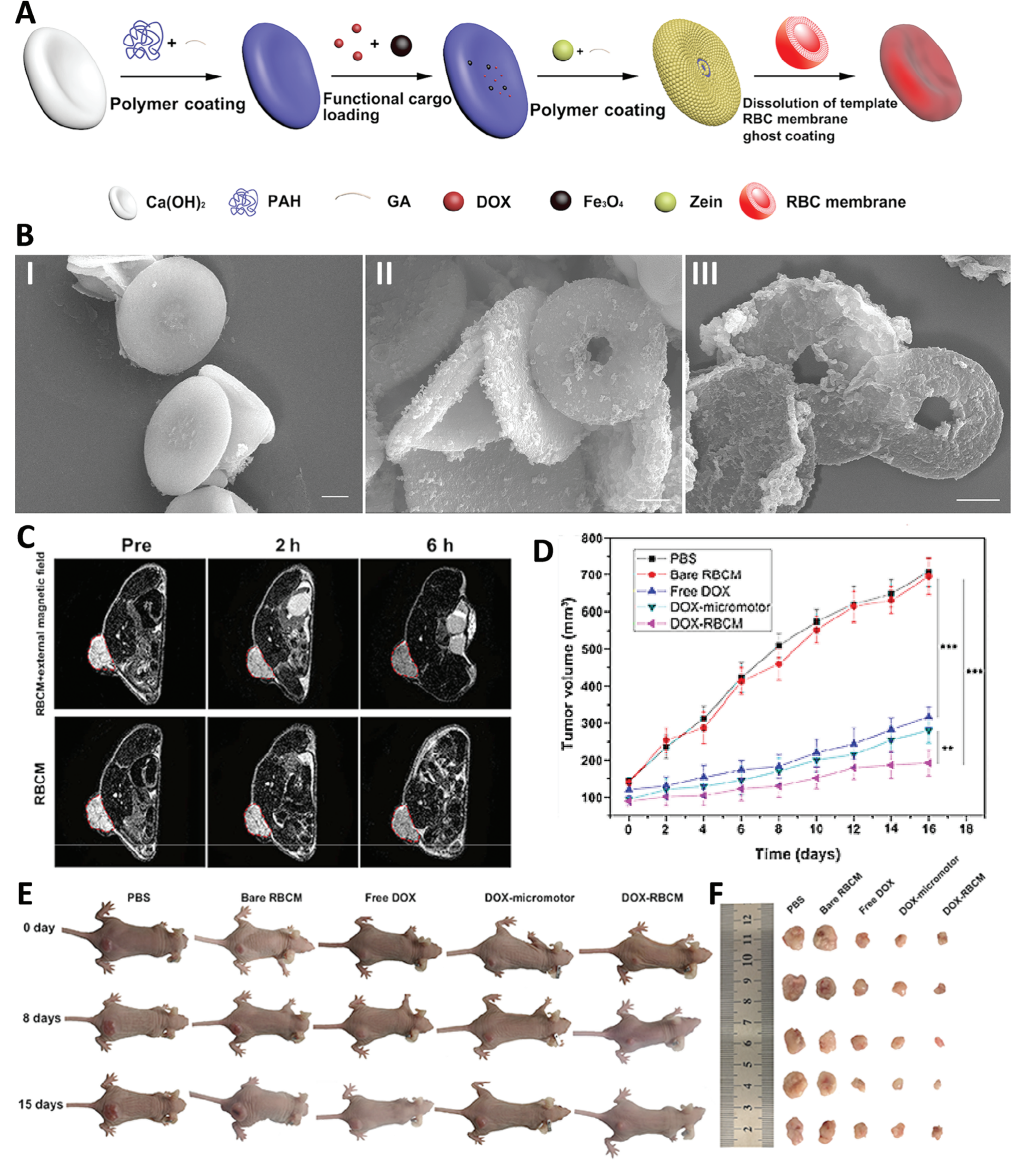
A) Schematic image of synthesizing RBCM. B) Morphology of template (I), RBCM (II), and RBCM hallow sphere (III). C) MR image of mice treated with RBCM at different times and in the presence/absence of an external magnetic field. D) Change in the volume of tumor exposed with different samples. E) Effect of different treatments on Mice with tumor for 15 days. F) *Ex vivo* results of different treatment on size of tumor after different times. Reproduced with permission.^[^ ^45^ ^]^ Copyright 2022, American Chemical Society.

In another study, a type of red blood cell‐mimetic micromotor (RBCM) was fabricated using layer‐by‐layer method for drug delivery and imaging guided therapy. To this aim, Ca(OH)_2_ microparticles with discoidal shape were used as template and covered with a polymeric shell, polyallylamine (PAH), and glutaraldehyde (GA). Then, this core‐shell microbot was then functionalized with Fe_3_O_4_ nanoparticles and DOX, followed by the addition of Zein and GA. Finally, the template was removed and RBC membrane nanovesicles covered the fabricated hollow microcapsules. Presence of Fe_3_O_4_ nanoparticles in the structure of this microbot provided the capability of T_2_‐weighted MR imaging in a concentration dependent manner. Results of hemocompatibility tests showed no differences between the effect of fabricated microbot and normal conditions. Besides, the RBCM showed no toxicity effect on Human umbilical vein endothelial cells (HUVEC), even at high concentrations, confirming the biocompatibility of the fabricated robot. Moreover, presence of natural derived zein in the structure of these microparticles provided biodegradability properties. This microbot also showed pH dependent drug release pattern so that near to 80% of DOX was released in pH 5 within 72 h, while just 30% of drug was released at normal pH at the same time. Moreover, drug loaded RBCM showed significant cytotoxicity effect against cancer cells so that the viability of cells was reduced to less than 25% at the concentration of 20 µg ml^−1^. According to the results of in vivo test, presence of RBC shell enhanced the circulation time of RBCM. These compounds were accumulated inside the tumor tissue, as determined via MRI test, even if they were injected intravenously. Moreover, presence of external magnetic field had enhanced their accumulation in the targeted organ. Besides, drug loaded RBCM showed significant tumor suppressing effect, compared with other treatments, while had no significant effect on vital organs that confirmed its therapeutic performance (**Figure** **10**).^[^ ^45^ ^]^

Self‐assembled stem cells doped with polydopamine (PDA) coated magnetic particles (MSCSMs) microrobots were fabricated in another study with the ability to adopt to different microenvironment via self‐configuration changes. Results of this study showed that conjugation of magnetic nanoparticles to the stem cells did not affect their viability or differentiation property. Integration of these microrobots with endoscopic equipment led to the fabrication of endoscopy‐assisted magnetic actuation with a dual imaging system (EMADIS) with the ability of penetrating into the narrow and deep sites of body without directly contacting with harsh and complex fluidic environments. Besides, utilizing an external magnetic field led to the direct delivery of the fabricated microrobots to the targeted tissue. Interestingly, presence of endoscopic view and MRI and ultrasound (US) imaging techniques provided the capability of monitoring the delivery process.^[^ ^46^ ^]^

Besides imaging, biohybrid micro/nanorobots could be applied as sensors for the detection of a specific analyte. For instance, gold nanowires covered with the hybrid membrane of RBCs and platelets (PLs) were used as biohybrid fuel free nanorobots for the detection of pathogenic bacteria and their toxin. Presence of different functional groups on the surface of membrane provided the capability of detecting different compounds. They had the capability of attachment to different pathogens due to the presence of PL and neutralized them. Moreover, they could be used for rapid detection of pathogenic bacteria and for removing them from biological fluid.^[^ ^47^ ^]^

**Figure 11:**
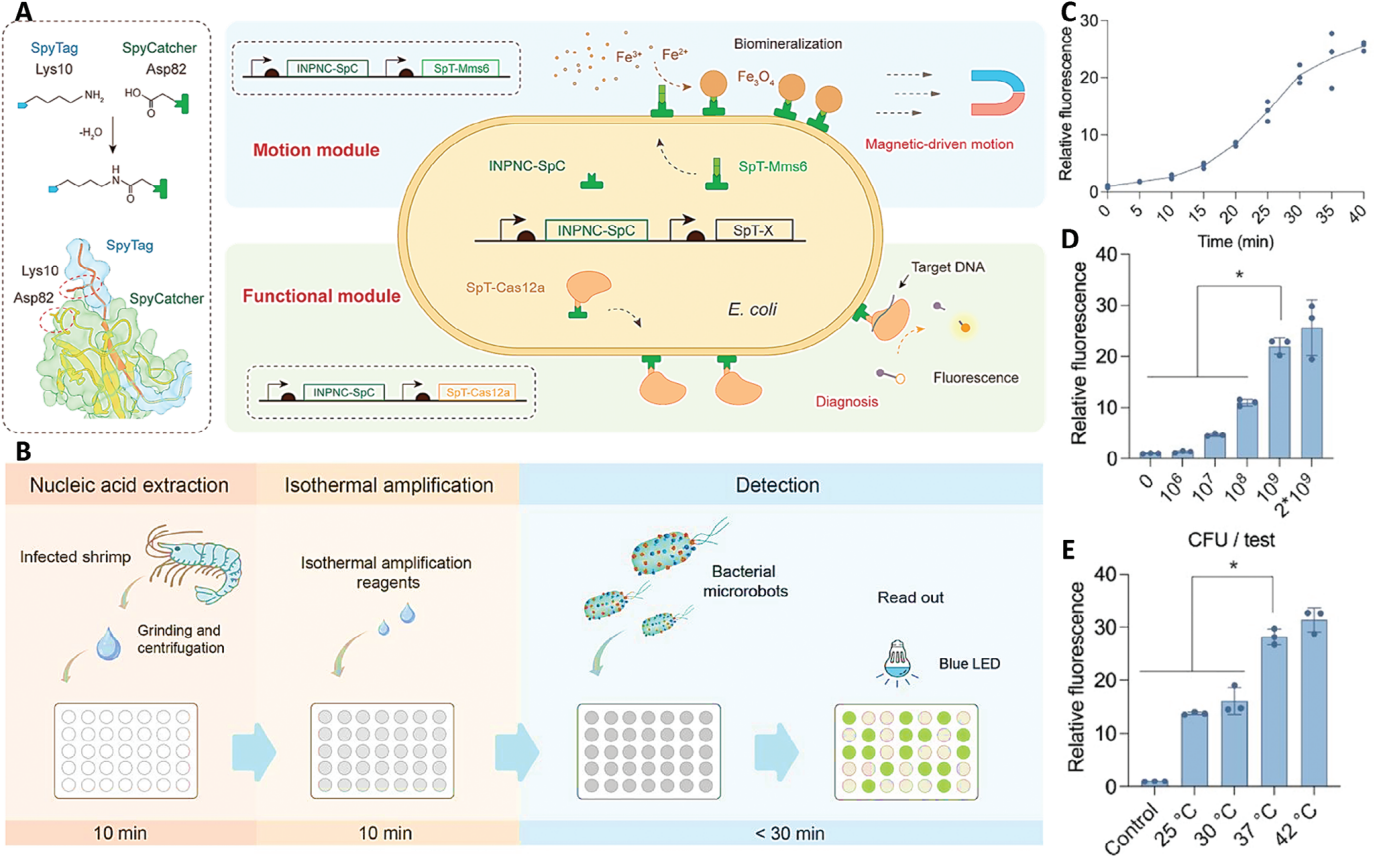
A) Schematic image related to the fabrication of bacterial microrobots used for the detection *Penaeus* viruses. B) Schematic image related to the detection process of the bacterial microrobot, encompassing nucleic acid extraction, isothermal amplification, and CRISPR‐based detection. Optimizing the C) detection time, D) microrobots concentration, and E) temperature of detection. Reproduced with permission.^[^ ^48^ ^]^ Copyright 2023, American Chemical Society.

Nucleic acid engineered *E. coli* were fabricated in a study by transforming specific genes in their DNA. Surface of these bacteria was engineered with magnetic nanoparticles and Cas12a (that used for the detection of aquatic pathogen nucleic acid). The surface of these microrobots was decorated with functional proteins that acted as the target‐locked and easily replaceable surface display module for testing the nucleic acid of shrimp viruses. This system allowed for the efficient preparation of microrobots in high throughput, ensuring stable performance. Interestingly, the bacterial microrobots could rapidly produce magnetic components by displaying the Mms6 protein on their surface, at room temperature and pressure, for magnetite biomineralization. Moreover, the robust nucleic acid detection module facilitated by CRISPR/Cas12a was presented on bacterial surfaces, equipping bacterial microrobots with a highly sensitive and specialized capability for swift diagnostics. The shrimp virus detection threshold was as low as a single‐digit copy per microliter, and the accuracy matched established gold standards. The fabricated microrobots exhibited reusability and high sensitivity with controlled motion through the magnetic navigation system, and their magnetization intensity surpassed that of naturally magnetic bacteria, measuring ≈18.65 emu g^−1^. They demonstrated excellent detection limits of ≈8 and 7 copies of µL^−1^ for decapod iridescent virus 1 (DIV1) and white spot syndrome virus (WSSV), respectively (**Figure** **11**).^[^ ^48^ ^]^

Biohybrid microrobots were also used for the detection of severe acute respiratory syndrome coronavirus 2 (SARS‐CoV‐2) in wastewater and its removal. Indeed, this virus could be alive in wastewater that could lead to spreading this virous by the water. In this study, *Chlamydomonas reinhardtii* (*C. reinhardtii*) algae were functionalized with angiotensin‐converting enzyme 2 (ACE2) receptor to fabricate fast motion biohybrid microrobots with long life span and the ability of attachment to the S1 subunit of the spike protein of SARS‐CoV‐2. They had the ability of movement in different media with high speed without any external energy. These microrobots not only detected the virus, specifically, but also removed them from the water in only 6 h.^[^ ^49^ ^]^

**Figure 12:**
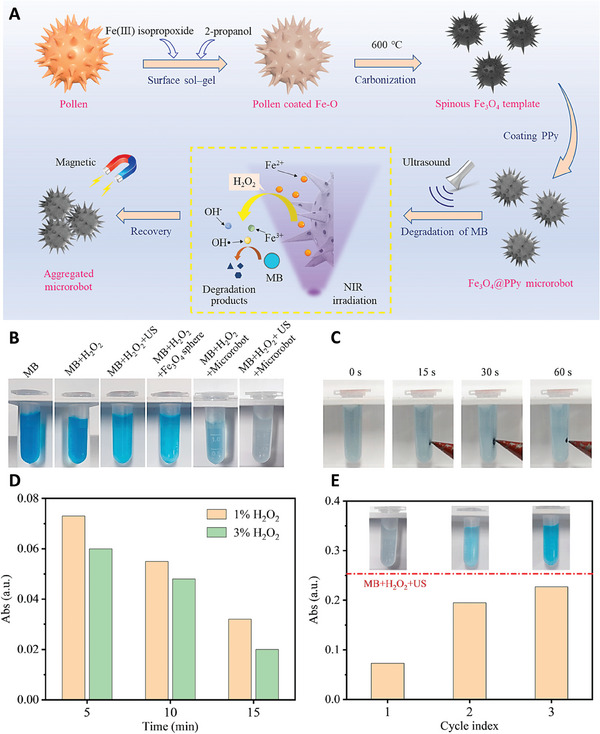
A) Schematic image related to the fabrication of Pollen based microrobot used for the detection of contaminations in water and remove them using ultrasonic and NIR irradiation. B) Effect of different treatment on destroying methylene blue (used as a model for contamination). C) Removing the microrobots from the water solution using external magnetic field. D) Determining the relationship between the anti‐pollutant effect of microrobot and the time and concentration of H_2_O_2_. E) Reusability of fabricated microrobot in removing methylene blue (MB). Reproduced with permission.^[^ ^50^ ^]^ Copyright 2023, Elsevier.

Sunflower pollen coated with Fe_3_O_4_ nanoparticles and polypyrrole (PPy) were used to fabricate microrobots with the ability of detection and remove organic pollutant. The fabricated microrobots showed different motions, including march‐like unific motion (MLM), typhoon‐like rotation toward the center gathered motion (TLM), and violent eruption‐like motion caused by local cavitation (ELM) under different ultrasonic frequency and sound pressure. This multimodal locomotion enabled microrobots to go through different dimensions of contaminated water and detected and attached to the pollutants. Utilizing ultrasound and NIR irradiation led to the release of Fe ions from these microrobots and destroying the pollutant through the activation of photo‐Fenton reaction. Moreover, presence of iron oxide nanoparticles in the structure of these nanoparticles enabled them to be collected in the presence of an external magnetic field and thus purified the water (**Figure** **12**).^[^ ^50^ ^]^ Some other types of biohybrid microrobots are summarized in **Table** **1**.

**Table 1:** Biomedical applications of biohybrid micro/nanorobots.

Micro/nanorobots	Targets	Outcomes	Refs.
DOX loaded ZIF‐8 nanoparticles coated *E. coli* MG1655	Drug delivery	‐ Protecting bacteria against enzymes ‐ pH responsive drug release behavior ‐ Strong interactions between bacteria and MOF ‐ Exhibiting chemotactic movement in response to the glucose gradient	[51]
ZIF‐8 functionalized *chlorella* loaded Fe_3_O_4_ nanoparticles	Drug delivery	‐ Exhibiting antibacterial activity resulted from release of Zn^2+^ (that could affect bacterial membrane, and also could affect bacterial nucleic acid) and collapse of ZIF (that led to release of imidazole ring of ZIF) ‐ Improving the antibacterial activity under magnetic actuation	[52]
Fe_3_O_4_ nanoparticles and DOX loaded pine pollen‐based micromotor (PPBM)	Drug delivery	‐ High adaptability with different media with prolonged lifetime ‐ Good targeting (in the presence of magnetic field) and swarm abilities ‐ Significant toxicity effect	[53]
DOX loaded urease‐powered nanomotors	Drug delivery	‐ Self‐propelling ability in the presence of urea ‐ Exhibiting time‐dependent enzyme activity, as well as pH dependent drug release ‐ Has urea dependent toxicity effect	[54]
Ultrasound responsive SonoBacteriaBot (DOX‐PFP‐PLGA@*EcM*)	Drug delivery	‐ Producing bacteriobot via fabricating ultrasound dependent PLGA nanodroplets loaded with DOX and perfluoro‐n‐pentane (PFP) and attaching it on the surface of E. coli MG1655 (EcM) ‐ Exhibiting targeting ability toward cancer cells, ultrasound imaging, and controlled drug release ‐ Inducing drug release in response to ultrasound stimulation	[55]
Paclitaxel loaded pH‐responsive peptide nanotubes (PNTs)‐coated microalgae	Drug delivery and anticancer activity	‐ Active targeted drug delivery via hiring the microenvironment features of cancer tissue ‐ Exhibiting fast movement related to the phototaxis behavior of *Chlamydomonas reinhardtii* (*C. reinhardtii*) algae ‐ Demonstrating pH responsive drug release behavior ‐ Inducing significant cytotoxicity effect against cancer cells in vitro and significant reduction in the size of tumor tissue in vivo	[56]
IRONSperm	Drug delivery and anticancer activity	‐ Improving the acoustic impedance of sperm ‐ Confirming biocompatibility of sperm ‐ Targeting ability in the presence of external magnetic field ‐ High drug encapsulation capacity and sustain release ability within 72 h	[57]
Urease immobilized Janus platelet micromotor (JPL‐motor)	Drug delivery, antibacterial, and anticancer activity	‐ Exhibiting effective movement in different biological fluid, especially in the presence of urea ‐ Do not affecting the adhesion properties of platelets ‐ Enhanced attachment on the surface of cancer cells and exhibiting significant anticancer activity ‐ Antibacterial activity resulted from their affinity to attach to the bacterial cells and release of antibacterial compounds	[58]
Magnetically steerable bacterial microrobots	Drug delivery and anticancer activity	‐ Surface functionalizing *E. coli* MG1655 with magnetic nanoparticles as well as DOX and ICG loaded liposome via utilizing streptavidin/biotin interaction ‐ Temperature responsive drug release resulted from NIR irradiation that led to increasing temperature ‐ Increasing temperature to ≈43 °C under NIR due to the presence of ICG ‐ Targeted delivery to cancerous tissue via using external magnetic field ‐ Localized active targeting drug delivery and responsive drug release	[59]
Magnetic nanoparticles@ mesenchymal stem cells (MSCs) biohybrid microbot (BHM‐MSCs)	Cancer therapy	‐ Targeted delivery of stem cells using rotating magnetic fields ‐ Loading magnetic particles inside the MSCs without affecting their viability, therapeutic performance, and migration abilities ‐ Exhibiting significant anticancer activity in the case of loading soluble tumor necrosis factor‐related apoptosis‐inducing ligand (sTRAIL) inside the BHM‐MSCs	[60]
DNA nanobot	Cancer therapy	‐ Fabrication of safe smart therapeutic compound targeted against nucleolin of cancer cells ‐ Delivering blood coagulation protease thrombin to induce thrombosis ‐ Inducing tumor necrosis and prevention of tumor growth	[61]
Anti‐HER2 aptamer (HApt) functionalized on tetrahedral framework nucleic acid (tFNA)	Cancer therapy	‐ Actively targeting HER2‐positive breast cancer cells ‐ Inducing HER2‐mediated endocytosis and lysosomal degradation ‐ Improved stability and prolonged the blood circulation time	[62]
CuS nanodots‐loaded biohybrid magnetic helical *Spirulina* derived microrobot (BMHM)	Anticancer and antibacterial activity	‐ Rapid temperature increased under NIR irradiation as well as stable photothermal effect ‐ Cost effective and easy fabrication ‐ Good targeting ability under external magnetic field ‐ Concentration dependent as well as irradiation time dependent antioxidant activity ‐ Good antibacterial activity	[63]
*Magnetospirillum magneticum* functionalized with light‐triggered indocyanine green nanoparticles	Anticancer activity	‐ Targeted delivery to the tumor site due to the presence of external magnetic field and internal hypoxia‐driven effects ‐ Ability of fluorescence and magnetic resonance imaging ‐ Photothermal therapy against cancer cells under irradiation with NIR laser ‐ Improved anticancer activity with nearly 100% inhibition within 22 days related to the photothermal effect	[64]
*Chlorella* microalgae decorated with Fe_3_O_4_ nanoparticles	Skeletal muscle tissue engineering	‐ Targeted delivery to the single C2C12‐derived myotube ‐ High biocompatibility ‐ Inducing hyperthermia in the presence of NIR that contracted the myotube via hiring a Ca^2+^‐independent method that involve direct actin–myosin interactions	[65]
Aptamer functionalized magnetic reduced graphene oxide/nickel/platinum nanoparticles (rGO/Ni/PtNPs) micromotors	Mycotoxin detection	‐ Relationship between the concentration of aptamer and signal intensity ‐ Rapid detection ‐ Reproducibility ‐ Selective and sensitive detection of fumonisin B1 (FB1) and ochratoxin A (OTA) ‐ Limit of detection (LOD) of about 0.7 and 4 ng mL^−1^ and linear range between 0.005–1 and 0.01–10 for FB1 and OTA, respectively ‐ Producing a reliable analytical tool for food‐safety monitoring	[66]
Magnetic helical nanomotors covered with platelet‐membrane‐cloaked	Pathogen and toxin detection and removal	‐ Prevention of biofouling of nanomotor in non‐treated biological media ‐ Rapid and selective detection of the Shiga toxin (produced by the *E. coli*) ‐ Protecting cells from toxins ‐ Rapid clearance of pathogens from media via direct or indirect interactions	[67]

Accordingly, the combined utilization of biological compounds like bacteria, stem cells, sperm, etc. and other types of organic and inorganic compounds, especially at the nanoscale, could create innovative machines for rapid targeted detection of disease or pollutants, attaching to them, destroying them (through releasing of drug or via hiring other treatment methods), tracking the healing process (via different imaging method), and finally eliminate them from the body, while have no toxicity effect. They could be considered as the state‐of‐the‐art strategies used in biomedical field, especially for the detection and treatment of disease, that could facilitate our ways toward achievement of personalized medicine, especially in the case of hard‐to‐treat disorders like cancer, which is regarded as the gold target of nanomedicine and pharmacology. The unique properties of these microrobots make them a promising alternative to other types of microrobots for future works in the field of minimally invasive surgery (MIS), which involves the insertion of a tethered tool from outside the body to the targeted site, typically equipped with a light source, small cameras, and mechanical devices (all in tiny sizes) for opening clogged vessels, cauterization, hyperthermia, biopsy, occlusion, electrical stimulation, injection, cutting, drilling, biomaterial removal, or addition at specific targets.

## 3 Challenges and Future Perspectives

Biohybrid micro/nanorobots have shown immense potential in various biomedical applications. These tiny robotic systems, often inspired by biological organisms, offer unique capabilities that can advance healthcare and biomedicine. Targeted drug delivery using biohybrid micro/nanorobots offers a promising approach to enhance the efficacy and precision of drug delivery in biomedicine. With their ability to navigate complex biological environments, specific targeting capabilities, and on‐demand drug release mechanisms, biohybrid robots have the potential to develop the field of drug delivery and improve patient outcomes. The combination of microsurgery and tissue engineering with biohybrid micro/nanorobots can open up new possibilities in the field of biomedicine. These tiny robots offer enhanced precision, control, and capabilities that can significantly improve surgical procedures and tissue regeneration techniques. In addition to therapeutic interventions, biohybrid robots can also be applied for diagnostic purposes. They can be equipped with imaging modalities such as fluorescence imaging or MRI to provide real‐time visualization and accurate assessment of disease states. Biosensing and monitoring applications of biohybrid micro/nanorobots hold immense promise in the field of biomedicine. Their ability to perform targeted biosensing, real‐time monitoring, and non‐invasive procedures offers new opportunities for early disease detection, personalized medicine, and improved patient care. Some biohybrid microrobots can be controlled remotely using external magnetic fields or other stimuli. This allows for non‐invasive manipulation and navigation within the body, further reducing the need for invasive procedures. While minimally invasive procedures with biohybrid microrobots hold great promise, there are still challenges to overcome. These include ensuring biocompatibility, addressing potential immune responses, and optimizing the control and navigation systems. Herein, we discussed about important challenges in areas such as biocompatibility, manufacturing, power and control, scalability, navigation and localization, stability, regulatory hurdles, and clinical translation:

### 3.1 Biocompatibility

Ensuring the compatibility of biohybrid micro/nanorobots with the biological environment is crucial. The materials used and the potential immune response need to be carefully considered to avoid adverse reactions or rejection. The materials used in constructing biohybrid micro/nanorobots must balance structural integrity, biocompatibility, and functionality. Achieving optimal biocompatibility involves a multifaceted approach encompassing material selection, surface modifications, and biological testing protocols. To this end, biocompatible materials should be carefully chosen based on their ability to mimic the properties of biological tissues, resist degradation in physiological conditions, and promote cellular compatibility and adhesion. Surface modifications, such as the incorporation of bioactive molecules or coatings, with the aim of enhancing the interaction between the biohybrid micro/nanorobots and the biological environment, and ensuring minimal interference with normal cellular functions, is another approach in this context.^[^ ^19^, ^68^ ^]^ Besides, future research should explore the development of smart materials with dual functionalities; those that not only maintain structural stability but also actively participate in the therapeutic process.

### 3.2 Power and Control

Providing a reliable power source and precise control mechanisms for biohybrid micro/nanorobots remains a challenge. Overcoming limitations in power supply and achieving real‐time control are areas of active research. The successful operation of these tiny devices hinges on the efficient delivery of power to propel their movements and precise control mechanisms to navigate through complex biological environments. One major challenge lies in determining the most suitable power sources for biohybrid micro/nanorobots that are both compact and capable of providing sustained energy for extended periods. Integrating power systems, such as micro‐batteries, micro fuel cells, or external magnetic fields, while ensuring minimal interference with biological processes, requires innovative engineering solutions. Control mechanisms for biohybrid micro/nanorobots pose another hurdle, necessitating robust strategies to steer and manipulate their movements with precision. Developing control algorithms that respond to external stimuli or internal signaling within biological systems is essential for directing the biohybrid devices toward specific targets or functions. The integration of sensory feedback systems to monitor and adjust the behavior of biohybrid micro/nanorobots in real‐time adds complexity to achieving precise control. Furthermore, coordinating power delivery with control mechanisms to enable autonomous operation and responsive behavior in dynamic biological environments poses a formidable challenge. Balancing the energy requirements for locomotion, sensing, and computation while ensuring seamless coordination between power and control systems demands innovative design approaches and advanced technologies.

### 3.3 Scalability and Manufacturing

Scaling down the fabrication process of biohybrid robots while maintaining their functionality and complexity can be challenging.^[^ ^69^ ^]^ Developing scalable manufacturing techniques is essential for practical implementation. One of the primary challenges in scalability is the translation of laboratory‐scale fabrication processes to mass production techniques. Many biohybrid micro/nanorobots are initially developed using specialized equipment and manual assembly methods which may not be viable for large‐scale manufacturing. Adapting these processes to high‐throughput manufacturing settings without compromising the performance or biocompatibility of the devices is a key challenge. Moreover, the integration of multiple components, including biological entities and synthetic materials, in biohybrid micro/nanorobots adds another layer of complexity to the manufacturing process. Coordinating the assembly of these diverse components in a controlled and reproducible manner while ensuring the functionality and biocompatibility of the final product poses a significant challenge. Research should focus on developing scalable, high‐throughput manufacturing techniques that allow for mass production without sacrificing quality or precision. Advancements in 3D and 4D printing technologies, which allow for the precise layering of materials at the nanoscale, could revolutionize how biohybrid robots are produced. 4D printing, in particular, offers the ability to create dynamic structures that change their configuration over time in response to external stimuli, providing a platform for developing more sophisticated robots that evolve with their environment. Nanomanufacturing techniques that utilize self‐assembly processes also show promise. Self‐assembling nanostructures could lead to the creation of biohybrid robots that autonomously form complex structures at the nanoscale, drastically reducing production costs and time. Exploring biofabrication techniques that leverage biological systems to build components, such as bacterial or yeast‐based systems, is another direction that could streamline the manufacturing process.

### 3.4 Navigation and Localization

Navigating biohybrid micro/nanorobots within the complex and dynamic environments of the human body, such as blood vessels or organs, presents significant challenges.^[^ ^18^ ^]^ Ensuring accurate localization and controlled movement is crucial for their effective use. One of the primary hurdles in navigation is the design of efficient propulsion mechanisms that allow biohybrid micro/nanorobots to navigate through diverse and often challenging biological environments. Overcoming obstacles such as viscosity, cellular barriers, and anatomical structures requires innovative propulsion strategies that enable controlled movement and maneuverability. For instance, utilizing different targeting or penetrating compounds and strategies could be a good choice for overcoming these challenges. Moreover, integrating sensors for environmental feedback and adaptive navigation capabilities enhances the ability of biohybrid micro/nanorobots to respond to changing conditions in real‐time.

Localizing biohybrid micro/nanorobots within biological tissues or organs presents another significant challenge. Ensuring precise localization at the sub‐millimeter or nanoscale level is crucial for targeted drug delivery, tissue manipulation, or diagnostic procedures. However, the complex and dynamic nature of biological systems poses challenges in maintaining accurate positioning and tracking of these miniature devices. Developing robust localization techniques, such as utilizing external magnetic fields, ultrasound imaging, or fluorescence‐based tracking systems, can aid in overcoming this challenge. Notably, coordinating navigation and localization strategies to enable autonomous behavior and effective targeting of biohybrid micro/nanorobots in vivo adds another layer of complexity. Future research should focus on developing autonomous navigation systems using artificial intelligence (AI) and machine learning algorithms. AI‐driven systems could allow biohybrid micro/nanorobots to learn from their environment, adapt to real‐time conditions, and optimize their movement toward specific targets. For instance, integrating onboard sensors that gather real‐time data on pH, oxygen levels, or temperature could enable the robots to adjust their movement autonomously. Combining this approach with AI models capable of predicting obstacles or changes in the body's environment would significantly improve the precision and effectiveness of these devices. Additionally, research into wireless energy transfer methods, such as electromagnetic resonance or ultrasound‐based energy harvesting, could eliminate the need for onboard power sources, reducing the size and enhancing the longevity of these robots.

### 3.5 Stability

The lifespan of biohybrid microrobots may be limited by factors such as degradation of biological components or mechanical wear and tear, which can impact their long‐term functionality. One of the crucial challenges in maintaining stability lies in the design and materials used for constructing biohybrid micro/nanorobots. Balancing the structural robustness of the devices with the need for biocompatibility and flexibility is crucial to prevent mechanical failures or damage during operation. Moreover, the integration of biological components with synthetic materials adds complexity to ensuring the stability of the biohybrid systems, as the interactions between these components must be carefully engineered to prevent deterioration or loss of function. Another important challenge related to stability is the long‐term functionality of biohybrid micro/nanorobots within biological environments. Ensuring that these devices retain their propulsion capabilities, sensing abilities, and targeting precision over extended periods is essential for their effectiveness in biomedical applications. Factors such as biofouling, enzymatic degradation, and immune responses present challenges to the stability of biohybrid micro/nanorobots and require innovative solutions to mitigate potential risks. Notably, maintaining stability under dynamic physiological conditions, such as temperature variations, pH changes, and mechanical stresses, adds complexity to the operational durability of biohybrid micro/nanorobots. Developing protective coatings, encapsulation strategies, or self‐healing mechanisms can enhance the stability of these devices and prolong their functional lifespan in vivo. For instance, TPU is recognized for its flexibility and durability, making it suitable for creating flexible components or coatings for nanorobots and microrobots. This flexibility enhances their mobility and adaptability within the complex environment of the human body. TPU‐coated nanorobots can maneuver through tight spaces, overcome obstacles, and target specific tissues or cells more effectively. Its resilience to mechanical stress and stability under various physiological conditions make TPU an asset in improving the functionality and maneuverability of drug‐delivery microrobots. Both PLA and TPU offer unique advantages in the design and manufacture of nano/microrobots tailored for specific drug delivery applications, leveraging their respective properties to enhance performance and efficacy.^[^ ^70^ ^]^

### 3.6 Regulatory Hurdles

The development and clinical translation of biohybrid micro/nanorobots face regulatory challenges, as ensuring their safety and efficacy requires rigorous testing and approval processes. One of the crucial challenges is aligning the design, manufacturing, and testing processes of biohybrid micro/nanorobots with regulatory requirements stipulated by health authorities such as the FDA (Food and Drug Administration) or the EMA (European Medicines Agency). Demonstrating compliance with standards for biocompatibility, sterilization, materials safety, and performance characteristics necessitates comprehensive preclinical and clinical evaluations, as well as robust documentation of the device development process. Moreover, the classification of biohybrid micro/nanorobots as medical devices or combination products further complicates the regulatory pathway, as these devices may involve a combination of biological and synthetic components that fall into different regulatory categories. Understanding the classification criteria and navigating the regulatory pathways for novel biohybrid technologies require expertise in regulatory affairs and close collaboration with regulatory agencies to ensure compliance. In addition, the dynamic nature of regulatory guidelines and evolving standards for emerging technologies pose challenges in keeping pace with regulatory updates and adapting to changing requirements. Ensuring that biohybrid micro/nanorobots meet current regulatory expectations while anticipating future regulatory trends is essential for successful market entry and acceptance by healthcare stakeholders.

### 3.7 Clinical Translation

While preclinical studies have demonstrated the promise of biohybrid micro/nanorobots, their translation to clinical settings is still in its infancy. The process of translating biohybrid micro/nanorobots from laboratory research to clinical applications presents substantial challenges that must be addressed to ensure their successful integration into healthcare practices. Clinical translation involves rigorous testing, validation, and regulatory approval to demonstrate the safety, efficacy, and feasibility of biohybrid micro/nanorobots in real‐world medical settings.^[^ ^4b^ ^]^ One of the crucial challenges in clinical translation is the accumulation of robust preclinical data that supports the potential clinical benefits of biohybrid micro/nanorobots. Future research should aim to standardize protocols for preclinical testing, including the establishment of comprehensive in vitro and in vivo models that closely mimic human physiological conditions. These models should account for the complexity of human biology, including the immune system's response, tissue‐specific interactions, and long‐term degradation of the robots within the body. Developing standardized tests for biocompatibility, safety, and effectiveness will be crucial in advancing biohybrid robots from laboratory research to clinical trials.

Demonstrating the therapeutic impact and added value of biohybrid micro/nanorobots over existing treatments is crucial for gaining support from clinicians, regulatory agencies, and investors. Notably, the scalability and reproducibility of biohybrid micro/nanorobots for clinical use present challenges in manufacturing processes, quality control, and distribution logistics. Implementing Good Manufacturing Practices (GMP) and quality assurance measures to ensure the reliability and safety of biohybrid micro/nanorobots is critical for clinical translation success. In addition, navigating the complexities of clinical trials, patient recruitment, ethical considerations, and healthcare system integration poses challenges in the clinical translation of biohybrid micro/nanorobots. In clinical trial design, a phased approach is necessary to minimize risks. Indeed, the initial trials should focus on applications with lower risk, such as diagnostic imaging or biosensing, before moving to more invasive uses like drug delivery or surgery. Patient safety must be the foremost concern, and ethical considerations surrounding the long‐term presence of biohybrid robots in the human body should be rigorously addressed. Researchers must also collaborate closely with regulatory bodies, such as the FDA and EMA, to ensure the trials meet all necessary safety and efficacy requirements. Standardizing the classification of biohybrid robots within regulatory frameworks will streamline the approval process for these advanced technologies. Moreover, collaborative efforts between academia, industry, and healthcare providers will be critical in navigating regulatory hurdles and ensuring that the design of biohybrid robots aligns with clinical needs and patient care standards.

## 4 Conclusion

Biohybrid micro/nanorobots represent a cutting‐edge technology with vast potential in biomedicine. Their ability to combine biological and synthetic components opens up new avenues for diagnostics, drug delivery, tissue engineering, and surgical interventions. Biohybrid micro/nanorobots can be designed to navigate through complex biological environments, enabling targeted delivery of drugs, therapeutic agents, or even performing precise interventions at specific sites within the body. These robots can be applied for precise manipulation and interaction with cells, tissues, or even individual molecules, which can be beneficial for various biomedical applications. They have the potential to transform minimally invasive procedures by enabling access to hard‐to‐reach areas within the body, reducing the need for invasive surgeries and promoting faster recovery times. By integrating biological components with robotic systems, biohybrid micro/nanorobots can combine the advantages of both worlds, such as incorporating biological sensing capabilities or utilizing biological materials for improved biocompatibility.

While these robots hold great promise, there are still challenges to overcome, such as biocompatibility, power supply, manufacturing techniques, and control mechanisms. Ensuring that these biohybrid systems harmoniously interact with biological entities without triggering adverse responses or rejections poses a significant hurdle. Achieving the delicate balance between functionality and biocompatibility demands meticulous attention to the materials used, surface modifications, and overall design of the micro/nanorobots. In addition, the manufacturing process of these intricate biohybrid systems necessitates precision and scalability. Integrating biological components with synthetic materials in a controlled and reproducible manner requires advanced manufacturing techniques, such as microfabrication and 3D printing, while navigating the complexities of handling delicate biological entities. Moreover, the need to optimize the performance of biohybrid micro/nanorobots while maintaining biocompatibility standards adds another layer of complexity to the manufacturing process. By optimizing the design, materials, and protective measures of biohybrid micro/nanorobots, researchers can enhance their stability, reliability, and performance in biomedical applications, ultimately advancing the field of miniature robotic systems for healthcare innovations. Addressing the challenges of regulatory hurdles in biohybrid micro/nanorobots demands proactive engagement with regulatory authorities, adherence to best practices in quality assurance and regulatory affairs, and a thorough understanding of the global regulatory landscape for medical devices. By addressing these challenges early in the development process and integrating regulatory considerations into device design and testing protocols, researchers can streamline the regulatory approval process and accelerate the translation of biohybrid micro/nanorobots from the lab to clinical settings, thereby realizing their full potential in advancing healthcare solutions. Overall, overcoming these challenges demands interdisciplinary collaboration, innovative approaches to material science, and a deep understanding of biological interactions, ultimately paving the way for the development of safe and effective biohybrid micro/nanorobots for diverse biomedical applications.

## Conflict of Interest

The authors declare no conflict of interest.

## References

[1] H. Zhang , J. Tang , H. Cao , C. Wang , C. Shen , J. Liu , ACS Appl. Nano Mater. 2024, 7, 17151.

[2] M. Li , X. Hu , Y. Zhao , N. Jiao , Adv. Mater. Technol. 2023, 8, 2201928.

[3] a) L. Sun , Y. Yu , Z. Chen , F. Bian , F. Ye , L. Sun , Y. Zhao , Chem. Soc. Rev. 2020, 49, 4043; 32417875 10.1039/d0cs00120a

[4] a) S. Preetam , P. Pritam , R. Mishra , S. Lata , S. Rustagi , S. Malik , Mol. Syst. Des. Eng. 2024, 9, 892;

[5] a) W. Xu , H. Qin , H. Tian , L. Liu , J. Gao , F. Peng , Y. Tu , Appl. Mater. Today 2022, 27, 101482;

[6] L. Wang , Z. Meng , Y. Chen , Y. Zheng , Adv. Intellig. Syst. 2021, 3, 2000267.

[7] L. Lu , H. Zhao , Y. Lu , Y. Zhang , X. Wang , C. Fan , Z. Li , Z. Wu , Adv. Healthcare Mater. 2024, 13, 2400414. 10.1002/adhm.202400414 38412402

[8] T. Chen , Y. Cai , B. Ren , B. Jurado Sánchez , R. Dong , Mater. Horiz. 2024, 11, 2772. 38597188 10.1039/d4mh00114a

[9] L. Gao , M. U. Akhtar , F. Yang , S. Ahmad , J. He , Q. Lian , W. Cheng , J. Zhang , D. Li , Acta Biomater. 2021, 121, 29. 33285324 10.1016/j.actbio.2020.12.002

[10] Y. Zhang , Y. Zhang , Y. Han , X. Gong , Micromachines 2022, 13, 648.35630115

[11] a) A. V. Singh , M. H. Dad Ansari , P. Laux , A. Luch , Expert Opin. Drug Deliv. 2019, 16, 1259; 31580731 10.1080/17425247.2019.1676228

[12] Z. Lin , T. Jiang , J. Shang , Bio‐Des. Manuf. 2022, 5, 107.

[13] Z. Wu , Y. Chen , D. Mukasa , O. S. Pak , W. Gao , Chem. Soc. Rev 2020, 49, 8088. 32596700 10.1039/d0cs00309c

[14] a) A. Halder , Y. Sun , Biosens. Bioelectron. 2019, 139, 111334; 31128479 10.1016/j.bios.2019.111334

[15] D. Jiao , Q. L. Zhu , C. Y. Li , Q. Zheng , Z. L. Wu , Acc. Chem. Res. 2022, 55, 1533. 35413187 10.1021/acs.accounts.2c00046

[16] J. Li , B. Esteban‐Fernández de Ávila , W. Gao , L. Zhang , J. Wang , Sci Robot 2017, 2, eaam6431. 31552379 10.1126/scirobotics.aam6431 PMC6759331

[17] S. Yu , Y. Cai , Z. Wu , Q. He , VIEW 2021, 2, 20200113.

[18] A. Elnaggar , S. Kang , M. Tian , B. Han , M. Keshavarz , Small Sci. 2024, 4, 2300211. 10.1002/smsc.202300211 PMC11935291 40212697

[19] Z. Li , T. Liu , X. Sun , Q. Zhou , X. Yan , Cell Rep. Phys. Sci. 2024, 5, 101979.

[20] B. Wang , K. Kostarelos , B. J. Nelson , L. Zhang , Adv. Mater. 2021, 33, 2002047. 10.1002/adma.202002047 33617105

[21] a) R. K. Sindhu , H. Kaur , M. Kumar , M. Sofat , E. A. Yapar , I. Esenturk , B. A. Kara , P. Kumar , Z. Keshavarzi , J. Drug Targeting 2021, 29, 822; 10.1080/1061186X.2021.1892122 33641551

[22] Y. Xu , Q. Bian , R. Wang , J. Gao , Int. J. Pharm. 2022, 616, 121551. 35131352 10.1016/j.ijpharm.2022.121551

[23] M. Li , J. Wu , D. Lin , J. Yang , N. Jiao , Y. Wang , L. Liu , Acta Biomater. 2022, 154, 443. 36243369 10.1016/j.actbio.2022.10.019

[24] Q. Chen , S. Tang , Y. Li , Z. Cong , D. Lu , Q. Yang , X. Zhang , S. Wu , ACS Appl. Mater. Interfaces 2021, 13, 58382. 34860489 10.1021/acsami.1c18597

[25] H. Zhang , Z. Li , C. Gao , X. Fan , Y. Pang , T. Li , Z. Wu , H. Xie , Q. He , Sci. Rob. 2021, 6, eaaz9519. 10.1126/scirobotics.aaz9519 34043546

[26] T. Studer , D. Morina , I. S. Shchelik , K. Gademann , Chem.–A Eur. J. 2023, 29, e202203913. 10.1002/chem.202203913 36757109

[27] D. Gong , N. Celi , D. Zhang , J. Cai , ACS Appl. Mater. Interfaces 2022, 14, 6320. 35020358 10.1021/acsami.1c16859

[28] X. Wang , J. Cai , L. Sun , S. Zhang , D. Gong , X. Li , S. Yue , L. Feng , D. Zhang , ACS Appl. Mater. Interfaces 2019, 11, 4745. 30638360 10.1021/acsami.8b15586

[29] N. Celi , J. Cai , H. Sun , L. Feng , D. Zhang , D. Gong , ACS Appl. Mater. Interfaces 2024, 16, 24341. 38687629 10.1021/acsami.4c02836

[30] a) S. Liu , C. Gao , F. Peng , Mater. Today Adv. 2022, 16, 100281;

[31] H. Huang , J. Li , C. Wang , L. Xing , H. Cao , C. Wang , C. Y. Leung , Z. Li , Y. Xi , H. Tian , Small 2023, 20, 2304088. 10.1002/smll.202304088 37939310

[32] D. Liu , T. Zhang , Y. Guo , Y. Liao , Z. Wu , H. Jiang , Y. Lu , ACS Appl. Bio Mater. 2022, 5, 5933. 10.1021/acsabm.2c00880 36384280

[33] H. Choi , B. Kim , S. H. Jeong , T. Y. Kim , D. P. Kim , Y. K. Oh , S. K. Hahn , Small 2023, 19, 2204617. 10.1002/smll.202204617 36354165

[34] C. Yan , K. Feng , B. Bao , J. Chen , X. Xu , G. Jiang , Y. Wang , J. Guo , T. Jiang , Y. Kang , Adv. Sci. 2024, 11, 2404456. 10.1002/advs.202404456 PMC11336935 38894569

[35] a) L. Sonntag , J. Simmchen , V. Magdanz , Molecules 2019, 24, 3410; 31546857 10.3390/molecules24183410 PMC6767050

[36] Y. Alapan , O. Yasa , B. Yigit , I. C. Yasa , P. Erkoc , M. Sitti , Ann. Rev. Control, Robot. Auton. Syst. 2019, 2, 205.

[37] G. Xing , X. Yu , Y. Zhang , S. Sheng , L. Jin , D. Zhu , L. Mei , X. Dong , F. Lv , Small 2023, 20, 2305526. 10.1002/smll.202305526 37798678

[38] H. Chen , Y. Li , Y. Wang , P. Ning , Y. Shen , X. Wei , Q. Feng , Y. Liu , Z. Li , C. Xu , ACS Nano 2022, 16, 6118. 35343677 10.1021/acsnano.1c11601

[39] D. Wu , Z. Zhao , H. Liu , K. Fu , Y. Ji , W. Ji , Y. Li , Q. Yan , G. Yang , Acta Biomater. 2023, 169, 477. 37532134 10.1016/j.actbio.2023.07.051

[40] C. Gao , Z. Lin , D. Wang , Z. Wu , H. Xie , Q. He , ACS Appl. Mater. Interfaces 2019, 11, 23392. 31252507 10.1021/acsami.9b07979

[41] M. Li , J. Wu , N. Li , J. Zhou , W. Cheng , A. Wu , L. Liu , N. Jiao , Adv. Funct. Mater. 2024, 34, 2402333.

[42] X. Yan , Q. Zhou , M. Vincent , Y. Deng , J. Yu , J. Xu , T. Xu , T. Tang , L. Bian , Y. X. J. Wang , Sci. Rob. 2017, 2, eaaq1155. 10.1126/scirobotics.aaq1155 33157904

[43] D. Zhong , W. Li , Y. Qi , J. He , M. Zhou , Adv. Funct. Mater. 2020, 30, 1910395.

[44] J. Wang , F. Soto , S. Liu , Q. Yin , E. Purcell , Y. Zeng , E. C. Hsu , D. Akin , B. Sinclair , T. Stoyanova , Adv. Funct. Mater. 2022, 32, 2201800.

[45] K. Hou , Y. Zhang , M. Bao , C. Xin , Z. Wei , G. Lin , Z. Wang , ACS Appl. Mater. Interfaces 2022, 14, 3825. 35025195 10.1021/acsami.1c21331

[46] B. Wang , K. F. Chan , K. Yuan , Q. Wang , X. Xia , L. Yang , H. Ko , Y.‐X. J. Wang , J. J. Y. Sung , P. W. Y. Chiu , Sci. Rob. 2021, 6, eabd2813. 10.1126/scirobotics.abd2813 34043547

[47] B. Esteban‐Fernández de Ávila , P. Angsantikul , D. E. Ramírez‐Herrera , F. Soto , H. Teymourian , D. Dehaini , Y. Chen , L. Zhang , J. Wang , Sci. Rob. 2018, 3, eaat0485. 10.1126/scirobotics.aat0485 33141704

[48] H. Chen , T. Zhou , S. Li , J. Feng , W. Li , L. Li , X. Zhou , M. Wang , F. Li , X. Zhao , ACS Appl. Mater. Interfaces 2023, 15, 47930. 37811735 10.1021/acsami.3c09690

[49] F. Zhang , Z. Li , L. Yin , Q. Zhang , N. Askarinam , R. Mundaca‐Uribe , F. Tehrani , E. Karshalev , W. Gao , L. Zhang , J. Am. Chem. Soc. 2021, 143, 12194. 34291944 10.1021/jacs.1c04933

[50] S. Yu , C. Liu , M. Sui , H. Wei , H. Cheng , Y. Chen , Y. Zhu , H. Wang , P. Ma , L. Wang , T. Li , Ultrason. Sonochem. 2024, 102, 106714. 38113586 10.1016/j.ultsonch.2023.106714 PMC10772293

[51] Y. Li , S. Tang , Z. Cong , D. Lu , Q. Yang , Q. Chen , X. Zhang , S. Wu , Mater. Today Chem. 2022, 23, 100609.

[52] B. Gu , J. Cai , G. Peng , H. Zhou , W. Zhang , D. Zhang , D. Gong , Colloids Surf. A 2024, 685, 133295.

[53] M. Sun , X. Fan , X. Meng , J. Song , W. Chen , L. Sun , H. Xie , Nanoscale 2019, 11, 18382. 31573587 10.1039/c9nr06221a

[54] A. C. Hortelão , T. Patiño , A. Perez‐Jiménez , À. Blanco , S. Sánchez , Adv. Funct. Mater. 2018, 28, 1705086.

[55] M. Du , T. Wang , R. Feng , P. Zeng , Z. Chen , Front. Bioengineer. Biotechnol. 2023, 11, 1144963. 10.3389/fbioe.2023.1144963 PMC9998949 36911192

[56] L. Ha , H. Choi , A. Singh , B. Kim , B. K. Kaang , Y. K. Oh , S. Kwang Hahn , D. P. Kim , Adv. NanoBiomed. Res. 2024, 2400042.

[57] V. Magdanz , I. S. Khalil , J. Simmchen , G. P. Furtado , S. Mohanty , J. Gebauer , H. Xu , A. Klingner , A. Aziz , M. Medina‐Sánchez , Sci. Adv. 2020, 6, eaba5855. 32923590 10.1126/sciadv.aba5855 PMC7450605

[58] S. Tang , F. Zhang , H. Gong , F. Wei , J. Zhuang , E. Karshalev , B. Esteban‐Fernández de Ávila , C. Huang , Z. Zhou , Z. Li , Sci. Rob. 2020, 5, eaba6137. 10.1126/scirobotics.aba6137 33022613

[59] M. B. Akolpoglu , Y. Alapan , N. O. Dogan , S. F. Baltaci , O. Yasa , G. Aybar Tural , M. Sitti , Sci. Adv. 2022, 8, eabo6163. 35857516 10.1126/sciadv.abo6163 PMC9286503

[60] R. A. Gundersen , T. Chu , K. Abolfathi , S. Gokcen Dogan , P. E. Blair , N. Nago , M. Hamblin , G. N. Brooke , R. M. Zwacka , A. Kafash Hoshiar , Cancer Nanotechnol. 2023, 14, 54. 37869575 10.1186/s12645-023-00203-9 PMC7615227

[61] S. Li , Q. Jiang , S. Liu , Y. Zhang , Y. Tian , C. Song , J. Wang , Y. Zou , G. J. Anderson , J. Y. Han , Nat. Biotechnol. 2018, 36, 258. 29431737 10.1038/nbt.4071

[62] W. Ma , Y. Zhan , Y. Zhang , X. Shao , X. Xie , C. Mao , W. Cui , Q. Li , J. Shi , J. Li , C. Fan , Y. Lin , Nano Lett. 2019, 19, 4505. 31185573 10.1021/acs.nanolett.9b01320

[63] D. Gong , N. Celi , L. Xu , D. Zhang , J. Cai , Mater. Today Chem. 2022, 23, 100694.

[64] J. Xing , T. Yin , S. Li , T. Xu , A. Ma , Z. Chen , Y. Luo , Z. Lai , Y. Lv , H. Pan , R. Liang , X. Wu , M. Zheng , L. Cai , Adv. Funct. Mater. 2021, 31, 2008262.

[65] L. Liu , J. Wu , B. Chen , J. Gao , T. Li , Y. Ye , H. Tian , S. Wang , F. Wang , J. Jiang , J. Ou , F. Tong , F. Peng , Y. Tu , ACS Nano 2022, 16, 6515. 35290021 10.1021/acsnano.2c00833

[66] Á. Molinero‐Fernández , A. Jodra , M. Moreno‐Guzmán , M. Á. López , A. Escarpa , Chem. – A Eur. J. 2018, 24, 7172. 10.1002/chem.201706095 29469987

[67] J. Li , P. Angsantikul , W. Liu , B. Esteban‐Fernández de Ávila , X. Chang , E. Sandraz , Y. Liang , S. Zhu , Y. Zhang , C. Chen , W. Gao , L. Zhang , J. Wang , Adv. Mater. 2018, 30, 1704800. 10.1002/adma.201704800 29193346

[68] J. Niu , C. Liu , X. Yang , W. Liang , Y. Wang , Front. Bioeng. Biotechnol 2023, 11, 1277964. 37781535 10.3389/fbioe.2023.1277964 PMC10539914

[69] J. Ye , Y. Fan , G. Niu , B. Zhou , Y. Kang , X. Ji , Nano Today 2024, 55, 102212.

[70] a) T. Das , S. Sultana , Future J. Pharmaceut. Sci. 2024, 10, 2.

